# The prophage-encoded transcriptional regulator AppY has pleiotropic effects on *E*. *coli* physiology

**DOI:** 10.1371/journal.pgen.1010672

**Published:** 2023-03-17

**Authors:** Naoual Derdouri, Nicolas Ginet, Yann Denis, Mireille Ansaldi, Aurélia Battesti

**Affiliations:** 1 Aix Marseille Université, Centre National de la Recherche Scientifique, Laboratoire de Chimie Bactérienne, Institut de Microbiologie de la Méditerranée, Marseille, France; 2 Aix Marseille Université, Centre National de la Recherche Scientifique, Plateforme Transcriptome, Institut de Microbiologie de la Méditerranée-, Marseille, France; Swiss Federal Institute of Technology Lausanne (EPFL), SWITZERLAND

## Abstract

Bacterial genome diversity is influenced by prophages, which are viral genomes integrated into the bacterial chromosome. Most prophage genes are silent but those that are expressed can provide unexpected properties to their host. Using as a model *E*. *coli* K-12 that carries 9 defective prophages in its genome, we aimed at highlighting the impact of genes encoded by prophages on host physiology. We focused our work on AppY, a transcriptional regulator encoded on the DLP12 prophage. By performing RNA-Seq experiments, we showed that AppY production modulates the expression of more than 200 genes. Among them, 11 were identified by ChIP-Seq as direct AppY targets. AppY directly and positively regulates several genes involved in the acid stress response including the master regulator gene *gadE* but also *nhaR* and *gadY*, two genes important for biofilm formation. Moreover, AppY indirectly and negatively impacts bacterial motility by favoring the degradation of FlhDC, the master regulator of the flagella biosynthesis. As a consequence of these regulatory effects, AppY increases acid stress resistance and biofilm formation while also causing a strong defect in motility. Our research shed light on the importance to consider the genetic interactions occurring between prophages and bacteria to fully understand bacterial physiology. It also highlights how a prophage-encoded transcriptional regulator integrates in a complex manner into the host regulatory network and how it benefits its host, allowing it to cope with changing environmental conditions.

## Author summary

Bacteriophages (or phages) are viruses that infect bacteria. Some of them have the ability to integrate their DNA into the bacterial chromosome, leading to the acquisition of new genes for bacteria. When expressed, these genes confer new properties to the host such as better fitness or increased stress resistance. Here, we study the function of AppY, a prophage-encoded transcriptional regulator in *Escherichia coli*. We found that AppY directly regulates 11 genes in *E*. *coli* and more broadly modulates the expression of more than 200 genes. These regulations lead to an increased survival of *E*. *coli* when the pH is low but also decrease bacterial motility and favor biofilm formation, a multicellular protective structure for bacteria. Overall, our work shows how a prophage-encoded protein modifies its bacterial host behavior to help coping with adverse conditions. Given the high number of viral genes present in bacteria and eukaryotic organisms, we anticipate a huge regulatory network between viral genes and those of the host, leading to major physiological changes. It is therefore crucial to uncover the new properties brought by viruses to bacterial hosts to combat the ever increasing number of bacteria resistant to many stresses including antibiotics.

## Introduction

Bacteriophages (or phages) can be classified according to their life cycles [1]. Virulent phages only perform a lytic cycle whereas temperate phages can adopt a lytic or a lysogenic cycle depending on the host physiology and the infection conditions. During the lysogenic cycle, the viral DNA integrates into the host chromosome as a prophage and is subsequently vertically transferred to the host progeny [2]. During evolution, prophage-encoded genes undergo all kind of mutations, integration and recombination events leading mainly to the loss of genes deleterious for the bacterium [3]. However, some genes called morons remain intact and retain functions providing benefits to their host [4]. Although the contribution of these morons to bacterial pathogenicity is an active field of research, considerably less data are available concerning their impact on bacterial fitness or stress resistance [5–7]. However, having a full picture of functional interactions linking prophages to their host is essential to fully understand bacterial physiology and assess the contribution of horizontally transferred functions.

Transcriptional regulators drive gene expression and consequently can have a major impact on bacterial physiology. Out of the roughly 300 transcriptional regulators encoded in the *E*. *coli* strain MG1655 genome, 9 are encoded by prophages [8–11]. Although the regulon of each of these prophage-encoded regulators has not been characterized in detail, data obtained so far suggest that a majority of them regulates the expression of prophage genes [11]. One noticeable exception is the transcriptional regulator AppY.

The gene *appY* is located in the defective lambdoid prophage DLP12 integrated into the *argU* tRNA gene of *E*. *coli* MG1655 [12,13]. DLP12 is the most prevalent prophage in *E*. *coli* strains and is also found in *Shigella* genomes [14]. The expression of *appY* is induced under anaerobiosis, phosphate and carbon starvations as well as during entry into stationary phase. Depending on the environmental conditions, its expression relies on the global regulators ArcA, H-NS, RpoS or the two-component system DpiA/B [15–18]. AppY is a transcriptional regulator from the AraC/XylS family, which is one of the most represented family of transcriptional regulators in bacteria [19]. Regulators from this family contain a characteristic DNA binding domain composed of two helix-turn-helix motifs. Most of them also contain a second domain involved in dimerization and/or effector binding. Members of this family are usually involved in general metabolism, virulence or stress responses [19–21].

To date, the exact role of AppY in cell physiology remains elusive and only a few AppY targets have been identified. Indeed, AppY has been shown to induce the expression of two operons located on the host chromosome: the *hya* operon coding for the hydrogenase 1 and the *app* operon coding for the cytochrome bd-II oxidase [12,15,16,22–25]. More recently, it has been shown that AppY overproduction leads to the stabilization of RpoS, the main sigma factor in stationary phase and the master regulator of the general stress response by a mechanism that has not been elucidated thus far [26,27]. Finally, a qualitative study has shown that AppY overproduction affects positively or negatively the level of more than 30 proteins in *E*. *coli* although none of these proteins were identified in that work [12].

Here, we aim to determine the contribution of AppY, a regulator acquired by horizontal gene transfer, to host physiology. By combining global approaches (RNA-Seq and ChIP-Seq) as well as forward genetics, we identified genes directly and indirectly regulated by AppY. We showed that AppY contributes to bacterial survival under low pH conditions, biofilm formation and repression of bacterial motility. We identified and characterized the regulatory pathways leading to each of these adaptive responses triggered by AppY. This study provides molecular insights into AppY integration into the *E*. *coli* regulatory network and highlights the importance of considering prophage-encoded genes to get a comprehensive picture of bacterial physiology.

## Results

### Global picture of the AppY regulon

**Fig 1:**
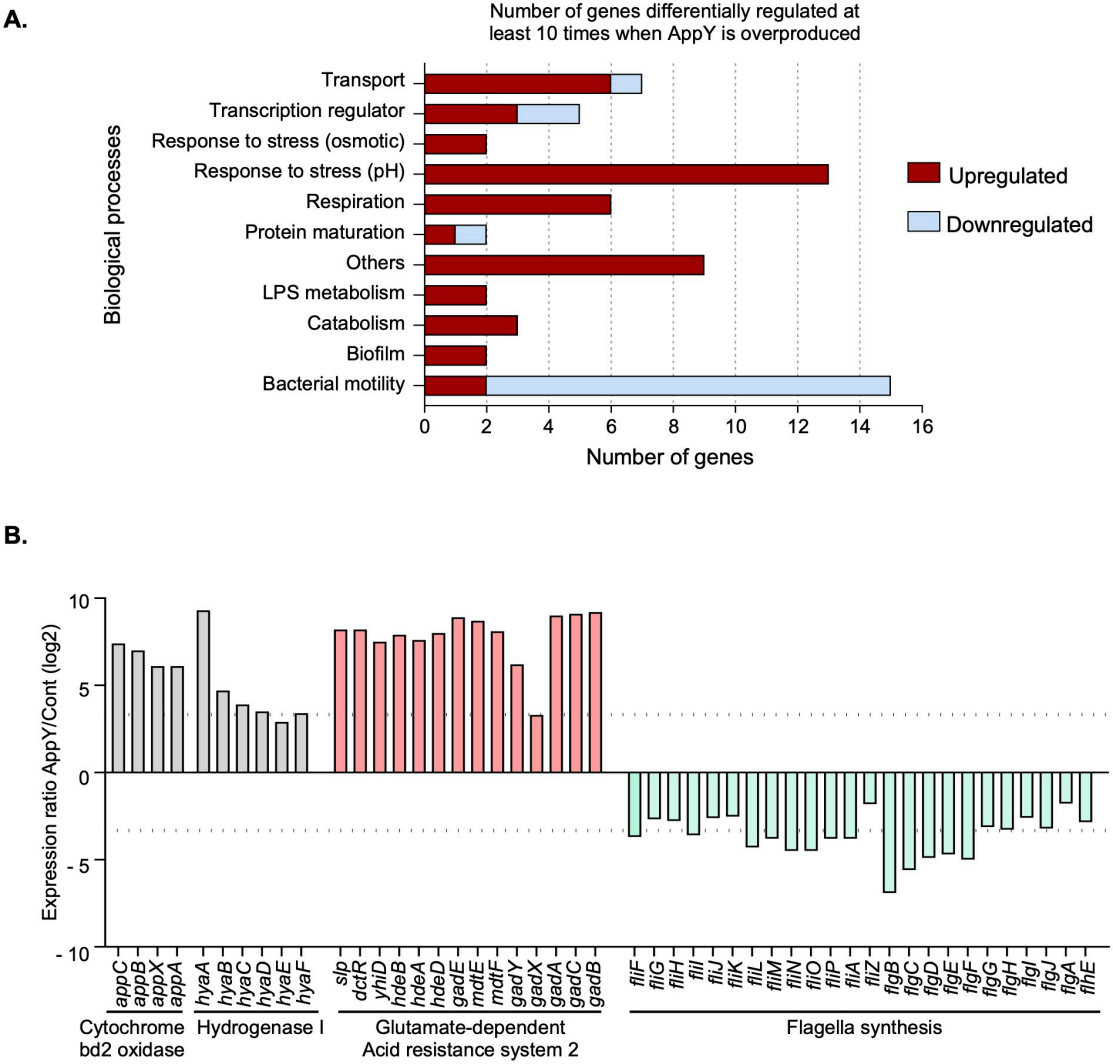
Identification of AppY regulon by RNA-Seq experiments. MG1655 Δ*rpoS* strain transformed with plasmids pQE80L or pQE80L-*appY* were grown to an OD_600_ ∼0.6 and *appY* expression was induced with 1 mM IPTG for 1 hour. RNA was purified, reverse-transcribed and sequenced. The data shown are representative of three independent experiments. A. Global overview of genes regulated at least 10 times by AppY. Genes up-regulated (dark red) and down-regulated (light blue) at least 10 times (Log2 Fold-Change ≥ 3.2) when AppY was overproduced were classified according to their biological functions. Categories were defined using the Gene Ontology numbers listed in S1 Methods. B. AppY mainly regulates genes involved in aerobic respiration (grey), acid resistance (pink) and flagellar synthesis (green). The dashed lines indicate 10-fold induction or repression.

No phenotype has ever been described for a strain deleted of *appY*, making it difficult to determine under which conditions AppY regulates its target genes or in which physiological process(es) it is involved. Therefore, we chose to overproduce AppY in order to define its regulon. As mentioned above, AppY overproduction stabilizes the master regulator RpoS, which itself regulates more than 500 genes in *E*. *coli* [27,28]. To avoid the identification of genes under RpoS control in our experiments, we overproduced AppY from an inducible plasmid in *E*. *coli* str. K12 substr. MG1655 strain deleted for *rpoS* (Δ*rpoS*). The same strain bearing the empty vector was used as a control. RNA-Seq experiments allowed us to identify more than 200 genes whose expressions were significantly modulated by at least a factor 4 upon AppY overproduction. In this study, we decided to focus on the 66 genes whose differential expressions are highly affected (at least 10-fold) (S1 Table). An overview of the AppY regulon is presented in Fig 1A where these 66 genes were classified based on their Gene Ontology numbers into 11 distinct biological processes. Among these genes, 43% belong to two main categories, *i*.*e*. response to stress (pH) and bacterial motility. About 75% of these 66 genes are upregulated and among those that are downregulated 77% are related to bacterial motility. This global picture indicates that the AppY regulon mainly concerns bacterial pH stress response as well as bacterial motility in addition to its previously known involvement into bacterial respiration [15,16,22,23,29].

In a closer view depicted in Fig 1B, we detailed the relative expression of genes involved in these three biological processes. We included genes below the 10-fold differential expression threshold (dotted lines) that belong to the same operon as genes differentially expressed above the 10-fold threshold. Firstly, we observed a massive induction of the two known AppY targets involved in respiration, the *hya* and *app* operons (containing 6 and 4 genes respectively, grey bars), therefore validating our strategy to identify the AppY regulon [30]. Secondly, 14 genes involved in acid stress resistance via the glutamate-dependent acid resistance system 2 (AR2), the major acid resistance pathway in *E*. *coli* [31], are upregulated (Fig 1B, pink bars). Finally, 24 genes, all of which are downregulated, are involved in flagella synthesis (Fig 1B, light green bars). Overall, these data suggest two unexpected roles for AppY in addition to its known involvement into respiration: it contributes to bacterial adaptation under low pH by inducing the glutamate-dependent response and it negatively regulates bacterial motility by down-regulating flagellar genes.

### Identification of genes directly regulated by AppY

RNA-Seq experiments provided a global overview of the physiological changes occurring in the cell when AppY was overproduced but did not provide information on AppY direct targets. To identify the genes that are directly regulated by AppY, ChIP-Seq experiments were performed. Briefly, the AppY protein with a 3Flag-tag at its C-terminal end was produced from an inducible plasmid in the Δ*rpoS* strain. Note that the 3Flag-tag did not affect AppY functionality as the flagged protein still induced the expression of *appC* and *hyaA* as measured with the transcriptional *gfp* fusions P_*appC*_*-gfp* and P_*hyaA*_*-gfp* (S1A Fig). After stabilization of the AppY-3Flag/DNA complexes using formaldehyde, chromosomal DNA was fragmented and cells lysed by sonication. AppY-3Flag/DNA complexes were then recovered by affinity purification using the 3Flag-tag and bound DNA was sequenced (see methods section for more details). To detect DNA-binding loci enrichment, the experiment was performed in parallel with three different controls: the empty vector, the untagged AppY and an AppY-3Flag mutant designed to affect its DNA binding capacity. Mutants affected in their DNA binding function have been well-characterized in other AraC-transcriptional regulators [32]. Based on sequence alignments, we identified a lysine residue at position 170 as potentially involved in AppY DNA-binding (S1B Fig). We used site-directed mutagenesis to generate the AppY_K170E_ mutant. AppY_K170E_ production was similar to that of the wild-type protein (S1C Fig) and did not allow the expression of the two transcriptional fusions used as positive controls P_*appC*_*-gfp* and P_*hyaA*_*-gfp* (S1A Fig). Furthermore, we observed that ChIP-Seq enrichment profiles were essentially identical with the empty vector or the vector bearing the *appY*_*K170E*_-3Flag construct. We thus confirmed that the K170 residue is critical for DNA binding.

**Fig 2:**
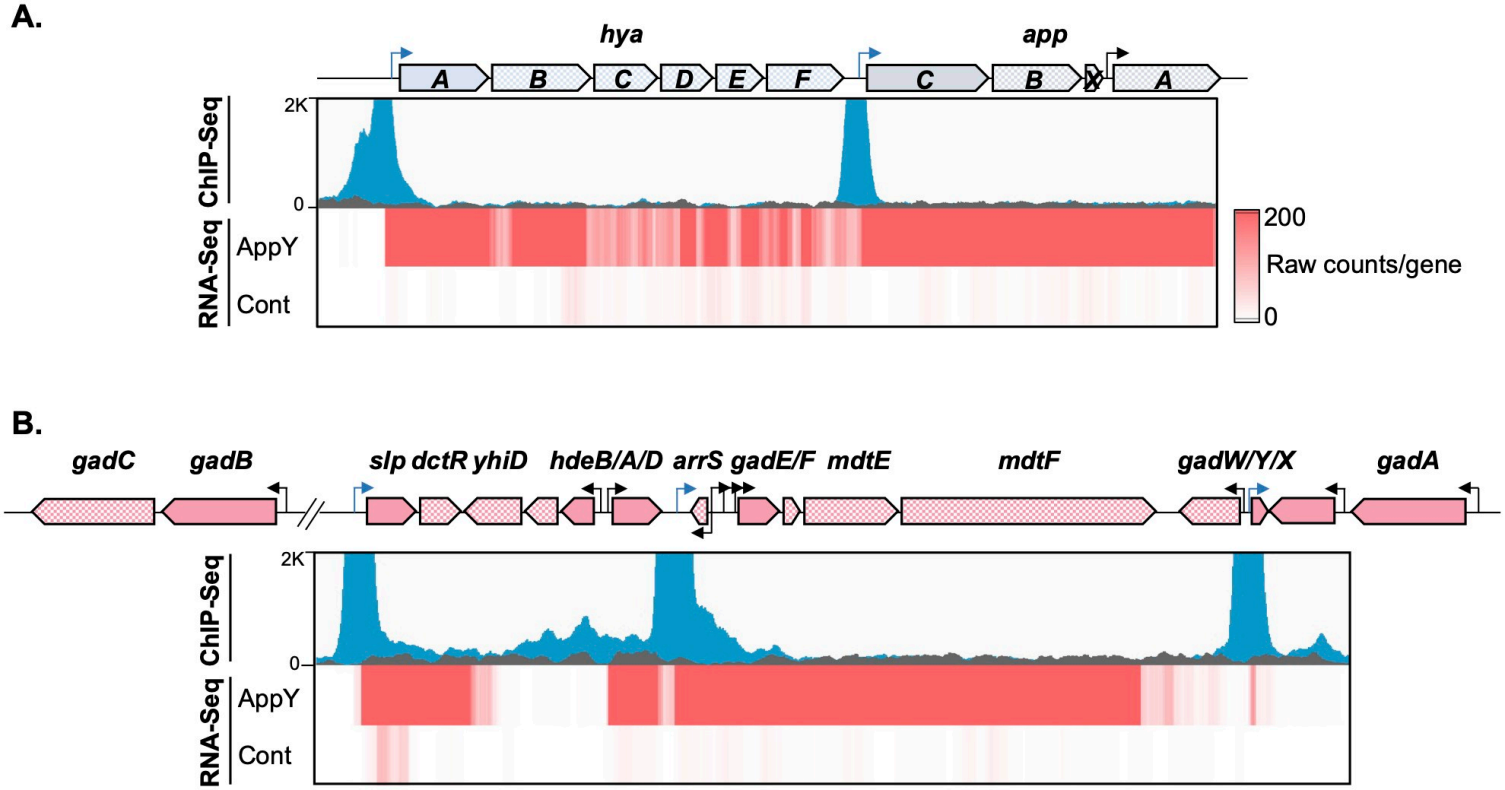
Identification of AppY binding sites by ChIP-Seq experiments. MG1655 Δ*rpoS* strain containing the plasmids pQE80L, pQE80L-*appY*-3Flag or *appY*_K170E_-3Flag were grown at 37°C to an OD_600_∼0.2 and *appY* expression was induced with 0.05 mM IPTG for 1 hour. The genetic loci, the ChIP-Seq data obtained with AppY-3Flag and AppY_K170E_-3Flag and the gene expression profiles obtained by RNA-Seq with AppY or the vector control are shown from the top to the bottom. In the genetic loci, genes with plain colour are located immediately downstream of a promoter; other genes are hatched. Peaks observed in ChIP-Seq with AppY_WT_ (blue) or AppY_K170E_ (black) are superimposed for comparison; the intensity of the red color in RNA-Seq panels represents the number of raw counts per gene. The data are representative of three independent experiments. A. Regulation of the *hya* and *app* operons. B. Regulation of the acid fitness island. The promoter regions where AppY binds are represented by blue arrows. *gadE* expression depends on four different promoters; AppY binds to the P3 promoter, the farthest from *gadE* start codon.

Peaks calling by MACS resulted in a list of 690 enriched DNA-binding loci (fold-change between 1.2 and 65.7) in the AppY-3Flag overproducing conditions compared to the control experiment (S2 Table). This list is identical whether the enrichment is computed with the untagged or the AppY_K170E_-3Flag mutant as a control, further confirming that the K170E mutant has indeed lost its capacity to bind DNA. We identified 22 significantly enriched chromosomal loci (at least 4 times) (Table 1). 86% of these binding regions are situated in known promoters or intergenic regions. Among them, 11 are upregulated at least 10 times when AppY is overproduced (highlighted in blue in Table 1). The detection of AppY binding in the promoter regions of *hyaA* and *appC* was in accordance with the RNA-Seq data and to our knowledge demonstrated for the first time that AppY directly regulates these operons (Fig 2A). Strikingly, no AppY binding site was identified in the promoter region of the genes involved in flagella synthesis that were heavily repressed in RNA-Seq experiments (Fig 1B, light green bars), suggesting an indirect regulation of these gene expression by AppY (Table 1). In contrast, AppY was clearly able to bind five promoter regions of genes involved in acid stress resistance: *slp*, *gadE*, *gadY*, *adiC* and *glsA* (Fig 2B and S2 Fig). Among them, *slp*, *gadY* and *gadE* are located in the acid fitness island and involved in the AR2 pathway. GadE is the master regulator of this pathway, suggesting that AppY directly regulates *gadE* expression with expected broad consequences on the AR2-dependent acid response [33–35]. In addition to its role in acid stress, the non-coding RNA GadY is also involved in biofilm formation [36–38]. We identified another direct AppY target involved in biofilm synthesis, the transcriptional activator NhaR [39]. These results suggest a supplementary regulatory role for AppY in biofilm formation. Overall, the ChIP-Seq results combined with those of the RNA-Seq suggest that AppY plays a key role in acid stress adaptation, biofilm formation and motility inhibition. In the following sections we explored the role of AppY in each of these pathways.

**Table 1:** AppY direct targets. Gene name, biological process, peak enrichment and peak absolute summit localization of AppY binding region on *E*. *coli* str. K12 substr. MG1655 chromosome (accession number U00096.3). In parenthesis are gene synonyms. "-" between two gene names indicates an intergenic binding locus. Biological processes follow the categories depicted in Fig 1A or one GO number description attached to the gene in Ecocyc when not included in Fig 1A. The differential gene expression computed in the RNA-seq experiment is indicated in the last column. When the fold change is ≥ 10 (log2 FC ≥ 3.2), genes are highlighted in blue (S1 Table). N.A means that the biological process cannot be attributed because the peak summit is between two genes.

Locus		ChIP-seq		RNA-seq
Gene	Biological process	Peak enrichment	Peak summit	Log_2_ FC
*gadE*	Response to stress (pH)	65.7	3657793	8.9
*gadY*	Response to stress (pH)	49.4	3664861	6.1
*appC (cbdX)*	Respiration	43.9	1037695	6
*slp*	Response to stress (pH)	43.1	3653884	8.2
*rrsG*	Translation	29.6	2731567	˂3.2
*Upp-purM*	N.A.	22.8	2620935	˂3.2
*hyaA*	Respiration	22.0	1031990	9.2
*acrZ*	Response to stress (cell envelope)	15.4	794574	˂3.2
*groS*	Protein folding	12.0	4370500	˂3.2
*glsA*	Response to stress (pH)	11.8	511563	4.8
*cdgI (yeaI)*	Cell motility	11.0	1871229	5.1
*yiiS*	Unknown	10.7	4112789	˂3.2
*adiC*	Response to stress (pH)	10.4	4337076	8.9
*ygiN*	Response to stress (pH)	9.8	3173053	˂3.2
*prc*	Proteolysis	9.7	1913928	3.4
*gtrA (yfdG)*	LPS metabolism	9.2	2467819	3.2
*nhaR*	Biofilm formation	9.1	18021	3.6
*fecI-insA-7*	N.A.	5.7	4518393	˂3.2
*dhaK-dhaR*	N.A.	4.9	1250957	˂3.2
*tolC*	Response to antibiotic	4.8	3177985	˂3.2
*yqcC*	Biofilm formation	4.8	2924287	˂3.2
*ygiC*	Nucleotide binding	4.6	3181040	˂3.2

### AppY directly and indirectly induces the expression of genes from the AR2 system

**Fig 3:**
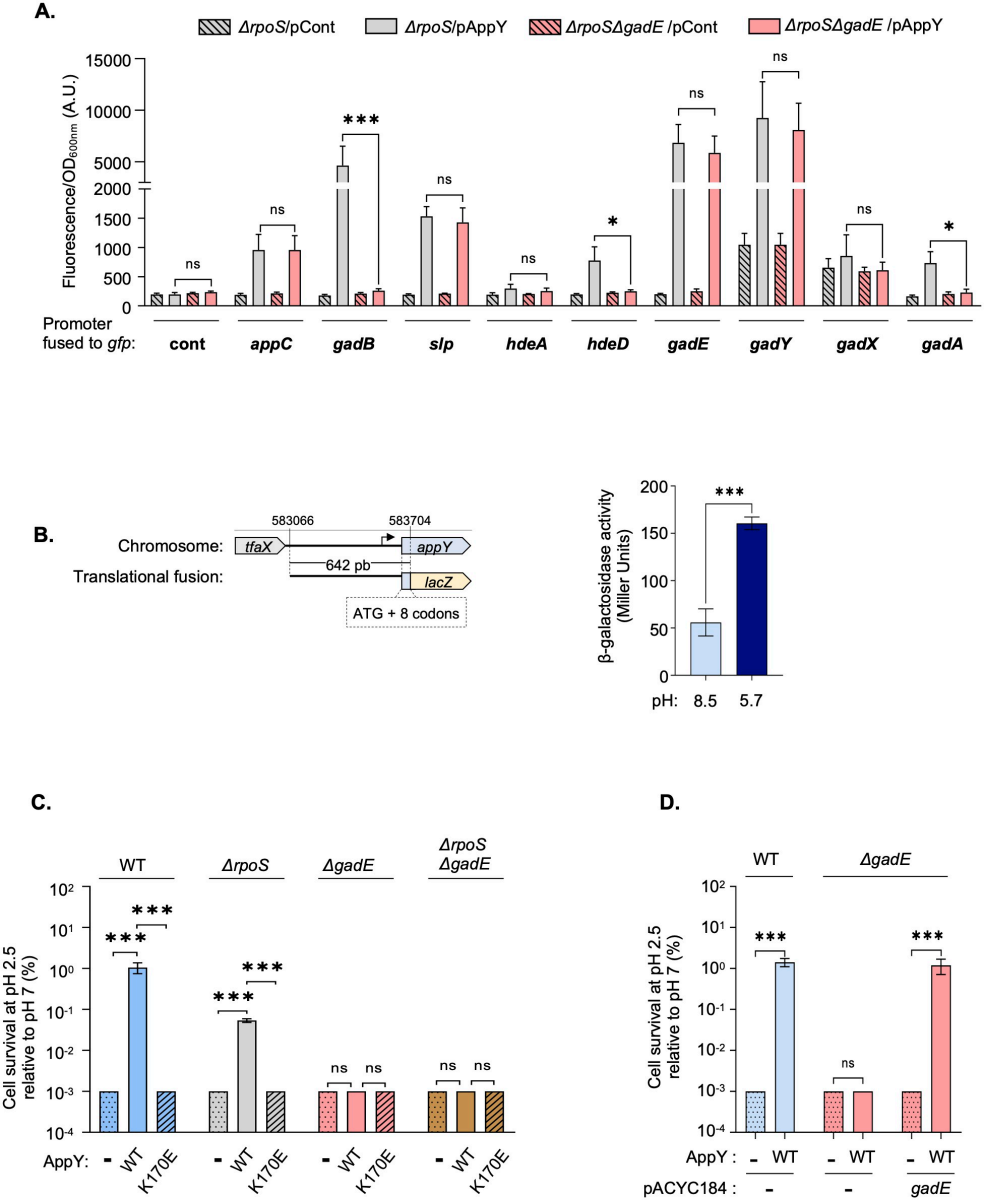
AppY contributes to acid stress response. A. *gadE*-dependent AppY targets. MG1655 strains deleted of *rpoS* (Δ*rpoS*, *grey*) or both *rpoS* and *gadE* (Δ*rpoS*Δ*gadE*, pink) and containing the pQE80L empty vector (pCont, hatched) or pQE80L-*appY* (pAppY, plain) were transformed with the indicated transcriptional *gfp* fusions (93). Relative fluorescence intensities were measured after ON growth at 37°C in LB plus 0.05 mM IPTG to induce *appY* expression. Data are means +/- standard deviation (n = 3). A.U., arbitrary units. Statistical significance calculations were performed using a two-tailed unpaired Student’s t test (ns, not significant; *, p-value < 0.05; ***, p-value < 0.001). B. Induction of *appY* expression under acid stress conditions. Left: Schematic representation of the *appY*-*lacZ* translational fusion. The genomic coordinates indicate the beginning and the end of the *appY* sequence fused to *lacZ* including the 9 first codons of *appY*. Right: The MG1655 strain carrying the chromosomal *appY-lacZ* translational fusion was cultured ON in LBK pH = 7, diluted 1:1000 into LB at pH 8.5 (light blue) or 5.7 (dark blue) and incubated at 37°C under aerobic conditions until OD_600_∼0.4. The activity of the *appY* fusion was determined using the Miller assay (89). The mean from three replicates is presented; the error bars represent the standard deviation (SD). Statistical significance calculations were performed using a two-tailed unpaired Student’s t test. (***, p-value < 0.001). C. AppY overproduction confers resistance to acid stress. WT (blue), Δ*rpoS* (grey), Δ*gadE* (pink) or Δ*gadE*Δ*rpoS* (brown) strains transformed with pQE80L (dotted), pQE-*appY*_WT_ (plain) or pQE-*appY*_K170E_ (hatched) were grown to OD_600_ = 1 in LB (pH 7.0) with 1 mM IPTG. Cells were diluted 1:200 into LB (pH 2.5) and incubated for 1 h at 37°C. Cells were spotted on plates to evaluate the number of cells that survive acid stress compared to the initial number of cells. Data are means +/- standard deviation (n = 3). Statistical significance calculations were performed using one-way ANOVA with Tukey’s multiple comparisons test (ns, not significant; ***, p-value <0.001). D. Complementation of MG1655 Δ*gadE* with *gadE* expressed under its own promotor. Strains MG1655 (blue) and *ΔgadE* (pink) were co-transformed with pQE80L (-, dotted) or pQE-*appY*_WT_ (WT, plain) and pACYC184 (-) or pACYC184-*gadE*. The experiment was carried out as described in 3.C. Data are means +/- standard deviation (n = 3). Statistical significance calculations were performed using one-way ANOVA with Tukey’s multiple comparisons test (ns, not significant; ***, p-value <0.001).

The majority of the genes positively regulated by AppY are part of the acid fitness island and contribute to the AR2 pathway (Figs 1 and 2; Table 1, S1 and S2 Tables) [34,35,40]. The regulation of this pathway is complex and involves a large number of regulators, including three transcriptional regulators from the AraC/XylS family: GadW, GadX and YdeO [31,34,41,42]. These three regulators modulate the expression of *gadE*, the master regulator of the AR2 pathway. The identification of *gadE* as a direct AppY target suggests that the induction of several genes from the AR2 pathway observed in the RNA-Seq experiment was probably GadE-dependent. To test this hypothesis, we used *gfp*-transcriptional fusions in strains deleted of *rpoS* only or both *rpoS* and *gadE* (Fig 3A). For genes in an operon, we used fusions containing the promoter region of the first gene of the operon (Fig 2B, plain pink genes in the genetic loci). Background levels of fluorescence were measured in strains containing the control plasmid (no promoter upstream of *gfp*) (Fig 3A, “cont” bars), or containing the fusions of interest in the absence of AppY overproduction (Fig 3A, hatched bars). The *appC* fusion was used as a positive control since we observed a direct AppY binding in its promoter region (Figs 2A and 3A). When AppY was overproduced in the strain deleted of *rpoS*, fluorescence levels were considerably increased for most of the fusions (from 4 to 34-fold; Fig 3A, grey bars). These results are consistent with the RNA-Seq data showing induction of these genes in the presence of AppY (Fig 1B). Only a mild effect was seen for *hdeA* and *gadX* (1.6 and 1.3-fold increase, respectively) whereas they were significantly upregulated in the RNA-seq experiments (181 and 9-fold, respectively). One possible explanation is that the increased level of *hdeA* and *gadX* mRNA measured with the RNA-Seq experiment in the presence of AppY is due to a post-transcriptional regulation. Indeed, in case this regulation involves sequences other than the promoter region or a small portion at the beginning of the coding sequence, it would be missed with the *gfp*-fusions. For *gadX*, this regulation could be dependent on the small non-coding RNA GadY that we identified as a direct AppY target and which is known to stabilize *gadX* mRNA by base-pairing with its 3’UTR (Table 1 and Fig 2B) [36]. In the strain deleted of *rpoS* and *gadE*, AppY overproduction led to the induction of *appC*, *slp*, *gadE* and *gadY*, the direct AppY targets we identified by ChIP-Seq (Fig 3A). However, AppY overproduction failed to induce the expression of *hdeD*, *gadB* and *gadA* in this genetic background. These results confirm that AppY directly induces *gadE* expression, which in turn activates its own regulon including *hdeD*, *gadB* and *gadA*. Altogether, these experiments identify AppY as a new player leading both directly and indirectly to the activation of the AR2 pathway.

### *appY* induction contributes to *E*. *coli* survival in acidic environment

Since AppY directly regulates *gadE* leading to the activation of the AR2 pathway, we could expect that *appY* itself is expressed under acidic conditions. Looking at the literature, we found two global studies reporting an induction of *appY* expression during a shift from pH = 8.5 to pH = 5.7; *appY* expression was further increased in the absence of oxygen [43,44]. Using a strain containing a chromosomal *appY-lacZ* translational fusion, we followed *appY* expression through beta-galactosidase assays under the growth conditions used in these studies. We measured a 2.8-fold increase in the activity of the fusion when the strain was grown at pH = 5.7 compared to pH = 8.5, validating the fact that *appY* expression was induced in acidic environment (Fig 3B). However, we did not detect a higher induction in the absence of oxygen (S3A Fig). The two-component system EvgA/EvgS has been shown to be involved in the sensing of acidic environment and activation of the AR2 system [45]. To determine whether EvgA/S and AppY belong to the same regulatory circuit, we measured the activity of the *appY-lacZ* fusion at pH = 5.7 or 8.5 in a strain deleted of *evgA* (S3B Fig). The absence of *evgA* did not affect *appY* induction at pH = 5.7, leading to the conclusion that *appY* is part of a regulatory pathway independent of the two-component system EvgA/S.

We then assessed the contribution of AppY to acid resistance under conditions known to activate the AR2 system. This system requires glutamate to function: in the absence of glutamate, the AR2 system is not active and the cell cannot survive at pH = 2.2 [33,46,47]. As a control, we used a strain deleted of *gadC* that codes for the transporter allowing glutamate entry in the cells. As expected, a strain deleted of *gadC* did not grow at pH = 2.2, with or without glutamate (S3C Fig). The WT and the Δ*appY* strains were unable to grow in the absence of glutamate but grew when glutamate was added without any significant difference (S3C Fig), indicating that in this experimental setup AppY is not essential for AR2 activation. We then postulated that if AppY was indeed involved in acid stress management, the benefit of AppY overproduction for the host could be an increased cell survival at low pH. To test this hypothesis, AppY wild-type or the K170E mutant was overproduced in the wild-type MG1655 strain; the cultures were then shifted from pH = 7 to pH = 2.5 for one hour and cell survival assayed by plating. Acid stress severely affected survival of the wild-type strain containing an empty vector after one hour at pH = 2.5 (Fig 3C, blue bars and S3D Fig). Strikingly, AppY overproduction massively increased cell survival by at least 100-fold under the same conditions. The increase in acid stress resistance was not observed with the AppY_K170E_ mutant that does not bind DNA (Fig 3C, hatched blue bars, and S3D Fig). These results confirm that AppY confers acid resistance and that this effect depends on its transcriptional regulator function. The effect of AppY overproduction was also tested in a Δ*rpoS* genetic background. In this context, we found that AppY overproduction still increased cell survival but 10-fold less than in a WT background (Fig 3C, compare WT (blue bars) with Δ*rpoS* (grey bars), and S3D Fig). This difference can be attributed to a higher sensitivity to acid stress of the strain lacking *rpoS*. To definitively link the positive effect of AppY on survival to the induction of the AR2 system, we repeated the same experiments in strains deleted of *gadE*. As expected, AppY overproduction did not increase bacterial survival in this genetic context (Fig 3C, pink and brown bars, and S3D Fig). However, when *gadE* was cloned together with its promoter region and co-transformed with pQE80L or pQE80L-*appY*_WT_ in the *ΔgadE* strain, it restored acid stress survival only in the presence of AppY (Fig 3D, pink bars, and S3E Fig). Overall, the data presented here demonstrate that AppY is involved in *E*. *coli* survival to acid stress, mainly by inducing *gadE* expression, hence the AR2 system.

### AppY overproduction increases biofilm formation *via* the simultaneous induction of *gadY* and *nhaR*

**Fig 4:**
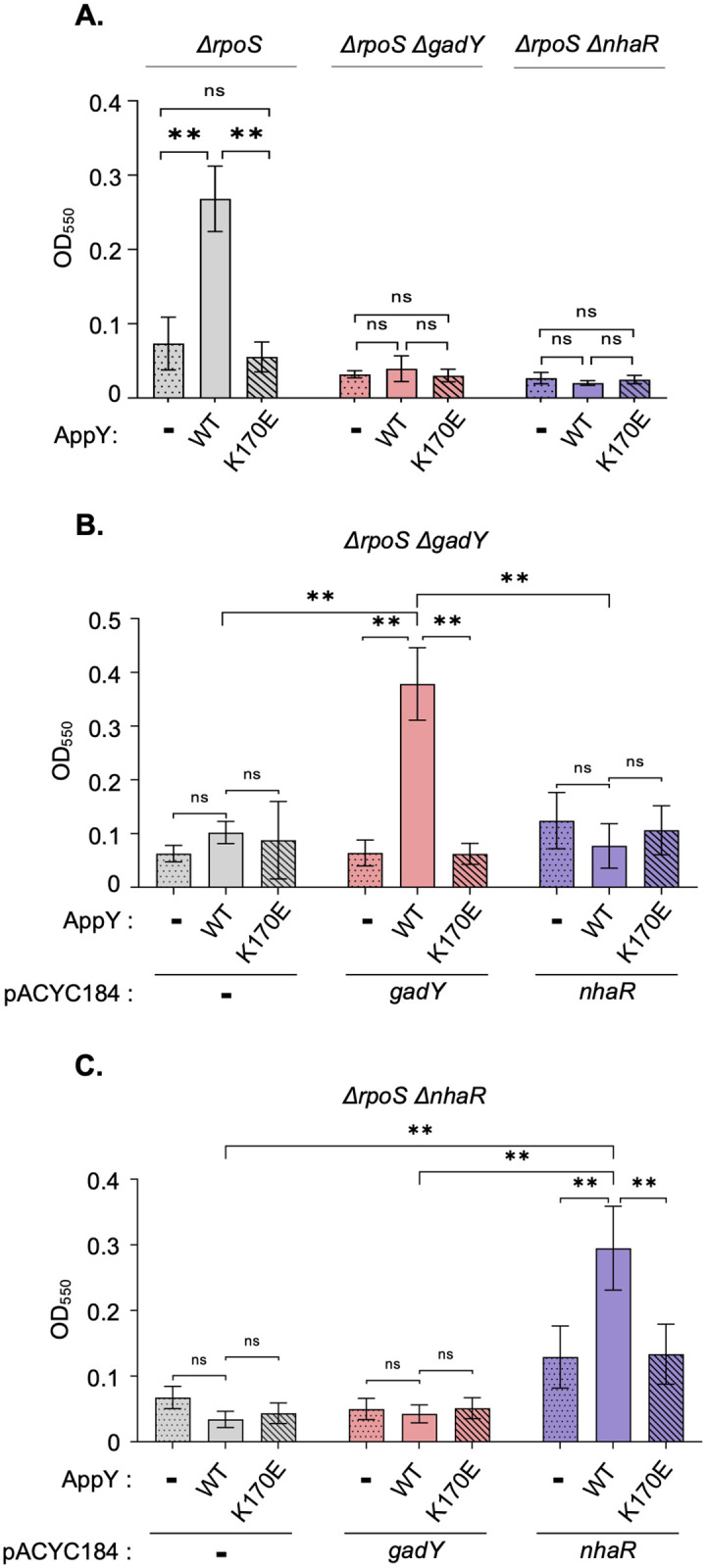
AppY favors biofilm formation through *nhaR* and *gadY* induction. The indicated strains were grown in LB with 0.5 mM IPTG at 30°C without shaking for 24 hours. Biofilm formation was visualized using crystal violet staining and quantified by measuring the optical density at 550 nm (OD_550_). The mean from three replicates is presented; the error bars represent the standard deviation (SD). Statistical significance calculations were performed using a two-tailed unpaired Student’s t test (ns, not significant; **, p-value < 0.01). A. Biofilm formation dependent on AppY, GadY and NhaR. MG1655 Δ*rpoS* (grey), MG1655 Δ*rpoS* Δ*gadY* (pink) or MG1655 Δ*rpoS* Δ*nhaR* (purple) were transformed with the pQE80L empty vector (-, dotted) or containing *appY* (WT, plain) or *appY* mutant (K170E, hatched). B. Complementation of MG1655 Δ*rpoS* Δ*gadY* with *gadY and nhaR* expressed under their own promoters. MG1655 Δ*rpoS* Δ*gadY* strain was co-transformed with the following plasmids: pQE80L empty vector (-, dotted), or containing *appY* (WT, plain) or *appY* mutant (K170E, hatched) and a pACYC184 construct (empty vector, -, grey), containing *gadY* (pink) or *nhaR* (purple). C. Complementation of MG1655 Δ*rpoS* Δ*nhaR* with *gadY and nhaR* expressed under their own promoters. MG1655 Δ*rpoS* Δ*nhaR* strain was co-transformed with a pQE80L construct (empty vector, -, dotted line), containing *appY* (WT, plain) or *appY* mutant (K170E, hatched) and a pACYC184 construct (empty vector, -, grey), containing *gadY* (pink) or *nhaR* (purple).

Out of the 11 direct AppY targets identified by ChIP-Seq, *gadY* and *nhaR* have been shown to be involved in biofilm formation through the up-regulation of the *pgaABCD* operon, responsible for the synthesis of the adhesin poly-ß-1,6-N-acetyl-D-glucosamine [38,39]. In order to test whether AppY overproduction led to biofilm formation, MG1655 Δ*rpoS* strain was transformed either with the pQE80L empty vector or with the same vector containing *appY* WT or the K170E mutant affected in DNA-binding. Cells were grown for 24 hours at 30°C and biofilm formation was quantified with crystal violet staining followed by OD_550_ measurement. This quantification clearly showed that AppY overproduction led to a 3-fold increase in biofilm formation (Fig 4A, grey bars and S4A Fig). AppY_K170E_ overproduction had no effect on biofilm showing that the observed phenotype is linked to the regulatory function of AppY (Fig 4A, grey hatched bars and S4A Fig).

To confirm that AppY acted on biofilm through GadY and NhaR, we deleted the corresponding genes in the MG1655 *ΔrpoS* background. AppY overproduction in *ΔrpoS ΔgadY* or in *ΔrpoS ΔnhaR* strains did not lead to biofilm formation suggesting that GadY and NhaR are both essential in this pathway (Fig 4A pink and purple bars respectively and S4A Fig). In order to quantify the contribution of these two regulators for biofilm formation, we cloned *gadY* and *nhaR* under their own promoters and co-transformed them with either pQE80L, pQE80L-*appY*_WT_ or pQE80L-*appY*_K170E_ in strains deleted of *gadY* or *nhaR*. In the presence of AppY, *nhaR* and *gadY* should be expressed from the plasmids and complement the biofilm formation defect of these two strains. In the MG1655 *ΔrpoS ΔgadY* strain, the plasmid expressing *gadY* showed no effect on biofilm when co-transformed with the control vector (Fig 4B, pink dotted bar and S4B Fig). However, the production of AppY led to the expression of *gadY* and restored biofilm formation (Fig 4B, plain pink bar and S4B Fig). As expected, no biofilm was observed with AppY_K170E_. Interestingly, in this genetic background, we did not observe an increase in biofilm with the plasmid expressing *nhaR*, even in the presence of AppY (Fig 4B, purple bars and S4B Fig). Altogether, this first set of experiments shows that NhaR alone is not sufficient to induce biofilm formation and that AppY contributes to biofilm formation through *gadY* induction. We then performed the same experiments in a MG1655 *ΔrpoS ΔnhaR* strain. In this background, GadY alone was not sufficient to induce biofilm formation, even when AppY was overproduced (Fig 4C, pink bars and S4C Fig). In the presence of *nhaR* and the control vector we observed a slight increase in biofilm formation; the same result was obtained with AppY_K170E_ (Fig 4C and S4C Fig). However, biofilm was fully restored only when both AppY and NhaR were produced at the same time (Fig 4C, plain purple bar). Overall, these data confirm that AppY favors biofilm formation by activating simultaneously the expression of the transcriptional regulator gene *nhaR* and the small RNA GadY.

### Characterization of the interaction between AppY and its DNA targets

**Fig 5:**
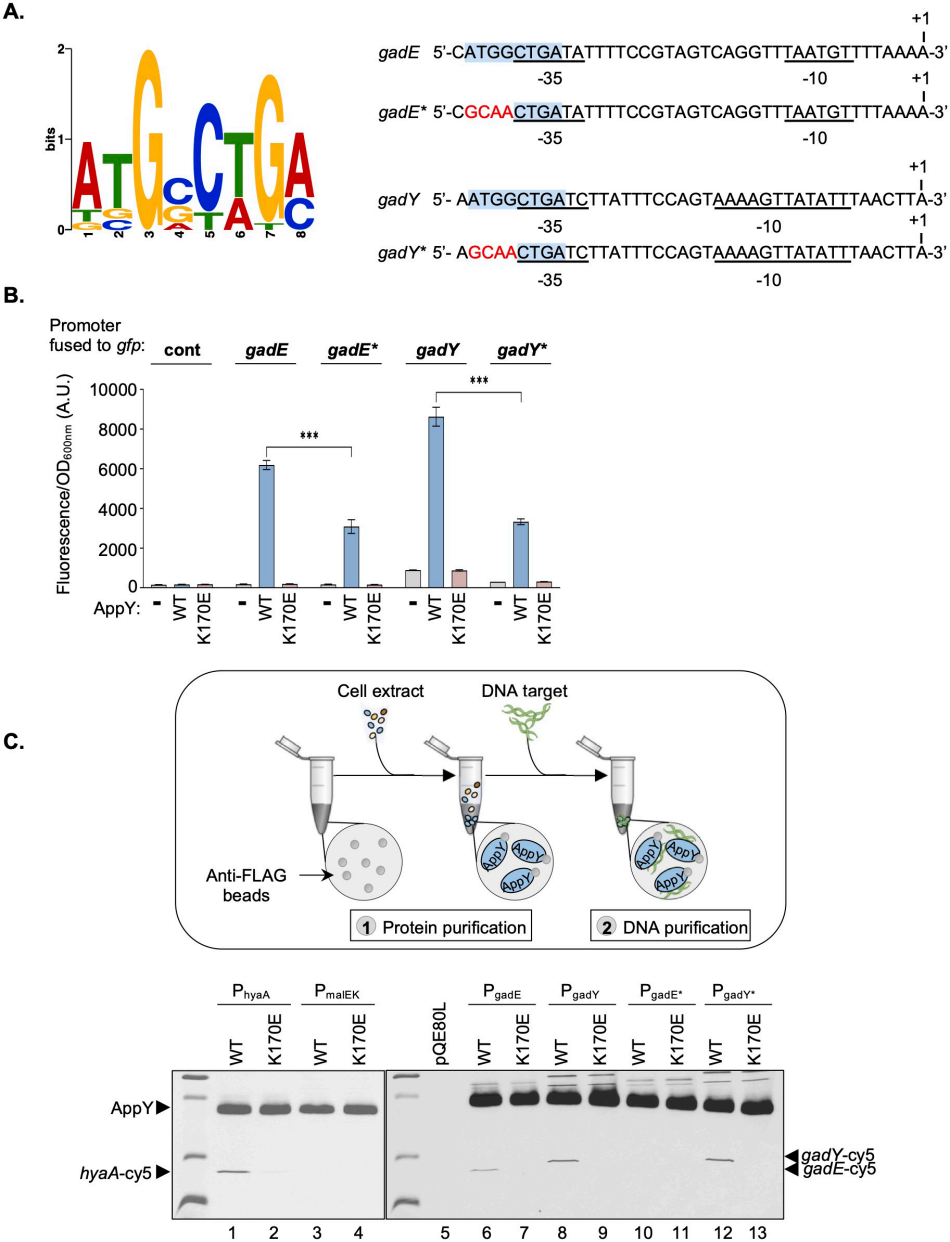
Identification of AppY binding sequence *in vivo* and *in vitro*. A. Left, Logo of the AppY DNA-binding motif consensus predicted by the MEME suite (ATGSCWGM). S: C or G; W: A or T; M: A or C. Right, *gadE* P3 promoter region and *gadY* promoter region. The -10 and -35 regions are underlined and the AppY DNA-binding motif is highlighted in blue. A new -35 sequence was defined for the P3 promoter of *gadE*. This sequence is at an optimal distance from the -10 hexamer and has a sequence closer to the consensus than the originally predicted -35 sequence (48). The mutated nucleotides in the variant promoter regions (*) are labelled in red. These mutations have been introduced in the *gfp*-transcriptional fusions or in the DNA fragments used in the pull-down assay. B. Mutations of the AppY binding site decrease the AppY-dependent induction of *gadE* and *gadY* transcriptional fusions. MG1655 Δ*rpoS* was co-transformed with the pQE80L empty vector (-, grey) or containing *appY* (WT, blue) or *appY* mutant (K170E, brown) and the indicated *gfp* fusions. Relative fluorescence intensities were measured after ON growth at 37°C in LB plus 0.05 mM IPTG to induce *appY* expression. Data are means +/- standard deviation (n = 3). A.U., arbitrary units. Statistical significance calculations were performed using a two-tailed unpaired Student’s t test (ns, not significant; ***, p-value < 0.001). C. AppY specifically binds to the promoter region of *hyaA*, *gadE* and *gadY in vitro*. Schematic representation of the pull-down assay. 1) Lysates containing AppY_WT_-3Flag, AppY_K170E_-3Flag or, as a control, a lysate made from cells containing the empty vector (pQE80L) were incubated with anti-Flag beads for purification. 2) Beads loaded with AppY_WT_-3Flag or AppY_K170E_-3Flag were incubated with DNA fragments labelled with Cy5 and corresponding to the promoter regions of *hyaA*, *gadE*, *gadY* or *malEK* as a control. AppY was detected with an anti-AppY antiserum and DNA fragments by fluorescence. The two revelations have been merged into a single image.

After demonstrating the physiological relevance of AppY positive regulation on some of its direct targets, we aimed at identifying a binding motif for AppY. We therefore subjected the DNA sequences of the 22 peaks enriched by at least four-fold identified in our ChIP-seq experiments (201 bp centered on the predicted absolute summit) to MEME Suite analyses. Although the binding site for most AraC/XylS family transcriptional regulators is 15 to 20 bp in length, we identified the 8 bp ATGSCWGM enriched motif in 20 out the 22 submitted sequences with a satisfactory E-value 6.8e-3 (Fig 5A). This motif was not found only in the enriched peaks upstream *acrZ* and *yiiS* (Table 1 and S2 Table). We noticed that the exact same motif overlapped the -35 sequence of P3, one of the four *gadE* promoters (blue arrow in Fig 2A situated downstream *hdeD*) and the -35 sequence of the *gadY* promoter. However, the distance between the identified motif and the -35 region was different between these two genes. Looking more closely at the *gadE* P3 promoter sequence, we identified a -35 element optimally positioned 17 bp upstream of the -10 hexamer and closer from the consensus than the one previously assigned [48]. Considering this new -35 element, the 8 bp motif was now located at the same position in *gadE* and *gadY* promoter regions, suggesting a unique AppY-dependent mechanism to induce transcription (Fig 5A). To confirm the role of this sequence in *gadE* and *gadY* AppY-dependent regulation, we mutated 4 of the 8 nucleotides in *gfp*-transcriptional fusions (labelled gadE* and gadY*) and monitored their activities (Fig 5B). Both mutated fusions displayed a lower activity than the WT fusions in the presence of AppY (decrease of a factor 2 and 2.6 for gadE* and gadY* respectively). This result shows that the identified sequence contributes to AppY-dependent regulation. To prove that AppY interacts with this sequence, we performed a pull-down assay (Fig 5C). Briefly, in a first step, AppY_WT_-3Flag or AppY_K170E_-3Flag were purified on anti-Flag beads. Then, PCR amplified Cy5-labeled promoter regions of *gadE* and *gadY* were incubated with the beads loaded with AppY WT or the K170E mutant. The same experiment was performed in parallel with the *hyaA* promoter region as a positive control and the *malEK* promoter region as a negative control since it was not identified as a direct AppY target. Moreover, the different DNA fragments were also all incubated together with beads in the absence of AppY to check for non-specific interactions. Using this technique, the *hyaA* DNA was retained on the beads in presence of AppY_WT_ whereas only a tiny amount was retained with AppY_K170E_ (Fig 5C, compare lines 1 and 2 and S5 Fig). As expected, no interaction was detected between AppY and the *malEK* promoter or with the different DNA fragments incubated with the beads only (Fig 5C, lines 3 to 5 and S5 Fig). An interaction was detected between AppY_WT_ and the promoter regions of *gadE* and *gadY* (Fig 5C, lines 6 and 8). This interaction is abolished in the presence of the K170E mutation, formally demonstrating that this mutant is affected in DNA binding (Fig 5C, lines 7 and 9). The same experiment was repeated with gadE* and gadY* DNA fragments mutated in the putative AppY binding motif. Mutation of this motif abolishes AppY interaction with the promoter region of *gadE* but not of *gadY* (Fig 5C, lines 10 to 12). The different sensibility of AppY to a mutation in the identified sequence suggests that this sequence is involved in AppY binding but does not represent the full AppY binding motif.

### AppY promotes the degradation of the master regulator FlhC, leading to a strong defect in bacterial motility

**Fig 6:**
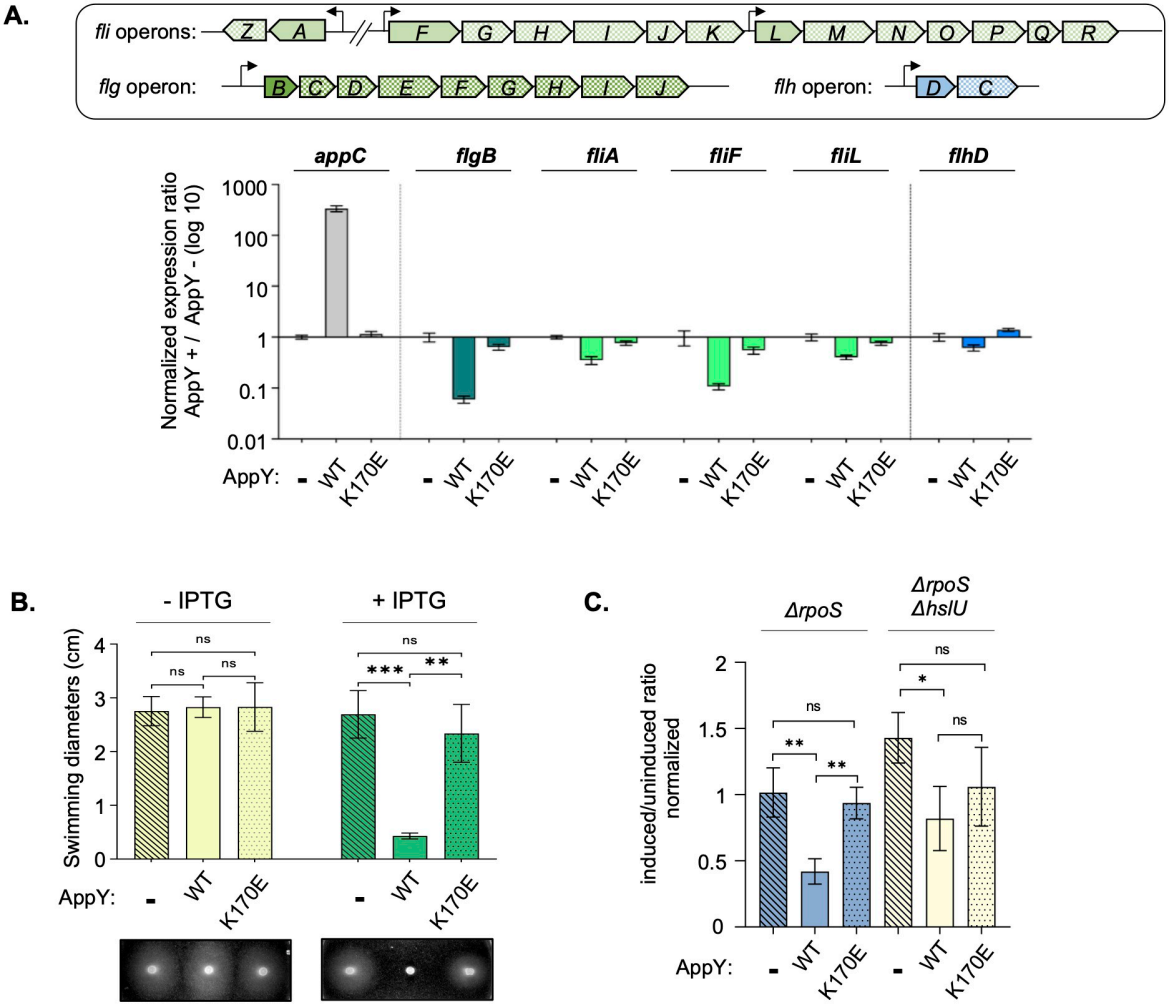
AppY indirectly reduces bacterial motility. A. Expression of flagellar genes is down-regulated by AppY overproduction. MG1655 Δ*rpoS* strain was transformed with the pQE80L empty vector (-) or containing *appY* (WT) or *appY* mutant (K170E). Cultures were grown to OD_600_∼0.5 and induced with 1 mM IPTG for 1 hour. RNAs were extracted from 10^9^cells and qRT-PCR experiments were performed. The genetic organization of flagellar genes is shown above the graph. Expression of the first gene of each operon (plain colour) was tested (*fliALF* in green, *flgB* in dark green, *flhD* in blue); *appC* (grey) is a control. Data are means +/- standard deviation (n = 3). B. AppY overproduction affects motility. ON cultures were adjusted to OD_600_ = 1 and 1μl was spotted on plates without (light green) or with IPTG (green). Plates were incubated at 30°C during 15 to 20 h and swimming diameters were measured. A representative picture of swimming is shown below the graph. Statistical significance calculations were performed using a two-tailed unpaired Student’s t test (ns, not significant; **, p-value < 0.01; ***, p-value < 0.001). C. AppY favors FlhC degradation by the HslUV protease. MG1655 Δ*rpoS* (blue) and MG1655 Δ*rpoS* Δ*hslU* (yellow) strains containing *flhC*-SPA on the chromosome were transformed with the pQE80L empty vector (-, hatched) or containing *appY* (WT, plain) or *appY* mutant (K170E, dotted). Cultures were grown to an OD_600_∼0.6 and induced with 1 mM IPTG at 37°C for 1 hour. FlhC levels were analysed by Western blotting using anti-Flag antibody. HtpG was detected as a loading control. Each value obtained for FlhC has been normalized on the loading control. Then the “Induced” samples were divided by the “Uninduced” samples for each condition. Statistical significance calculations were performed on these data using a two-tailed unpaired Student’s t test (ns, not significant; *, p-value < 0.05; **, p-value < 0.01).

According to the RNA-Seq experiments, AppY overproduction strongly down-regulates several genes involved in flagellar formation and motility (Fig 1 and S1 Table). However, the ChIP-Seq data showed no AppY binding site upstream of these genes suggesting that this regulation was indirect (Table 1 and S2 Table). To confirm the negative effect of AppY on the expression of flagellar genes, we quantified the level of expression of the first gene of each operon when AppY was overproduced using qRT-PCR (Fig 6A; plain arrows). The *appC* gene was used as a positive control since we have demonstrated that it is directly and positively regulated by AppY (Figs 2B and 6A). In agreement with the RNA-Seq data, expression of all of the *fli* and *flg* genes tested was reduced between 2.5 and 16-fold in the presence of AppY. This decrease in gene expression was not observed with AppY_K170E_ confirming that an intact DNA binding domain is needed to exert this repression (Fig 6A). We then assayed the consequences of this repression on *E*. *coli* motility. We transformed pQE80L-*appY*, pQE80L-*appY*_K170E_ or the control vector pQE80L into the MG1655 *ΔrpoS* strain and performed a classical swimming test on soft-agar plates. In the absence of inducer, all strains were equally motile (Fig 6B, light green bars). In contrast, in the presence of IPTG, AppY overproduction inhibited cell motility (Fig 6B, dark green bars). Here again, motility inhibition was not observed with the AppY_K170E_ mutant indicating that the DNA binding activity is required in this process (Fig 6B).

The global downregulation of a large number of genes involved in flagella synthesis suggests that FlhDC, the master regulator of this pathway, could itself be affected by AppY overproduction. However, neither the RNA-Seq nor the qRT-PCR experiments showed any significant decrease in *flhDC* expression when AppY was overproduced (Fig 6A, blue bars and S1 Table). Therefore, the repression of the *fli* and *flg* operons cannot be attributed to a direct AppY effect on *flhDC* transcription or mRNA stability. Interestingly, FlhD and FlhC have been previously shown to be actively turned-over by the Lon and ClpXP proteases in *Proteus mirabilis* and *Salmonella enterica* Typhimurium, respectively [49,50]. In order to follow the level of FlhDC in the presence of AppY, we fused a SPA-tag to FlhD and FlhC C-terminal extremities; for unknown reasons, this tag only led to FlhC detection. However, the absence of detection for FlhD when produced alone has already been described [50]. We quantified the level of FlhC, in the presence of the empty vector and observed a constant level of FlhC with or without IPTG (Fig 6C). When AppY was overproduced, a decrease in FlhC amount was visualized; this decrease did not occur with the AppY_K170E_ mutant (Fig 6C, blue bars). This result shows that AppY production favors FlhC degradation. Looking at our RNA-Seq experiments, we noticed that the expression of the *hslUV* protease was greatly enhanced in the presence of AppY (4.7 and 6-fold for *hslU* and *hslV*, respectively). In order to determine if this protease complex was responsible for FlhC degradation, we measured the FlhC level in strains deleted for *hslU*. This deletion led to FlhC-SPA stabilization even in the presence of AppY (Fig 5C, light yellow bars). Overall, the data presented here suggest that AppY production results in the degradation of FlhC, which is at least partially dependent on HslUV. This active degradation leads to a severe motility defect in *E*. *coli*.

## Discussion

**Fig 7:**
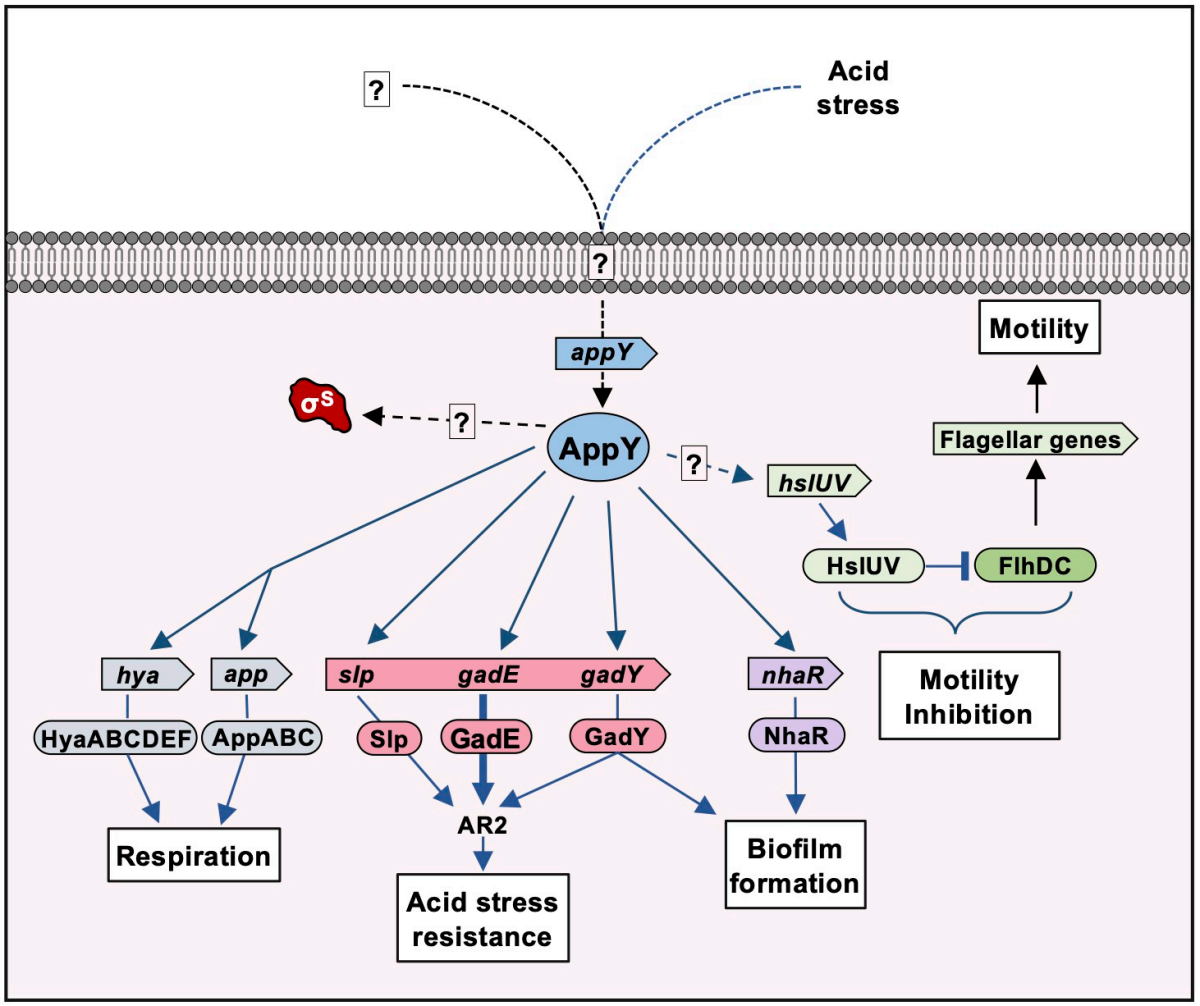
Pleiotropic effects of AppY on *E*. *coli* physiology. AppY activates several regulatory cascades leading to acid stress resistance, biofilm formation and motility inhibition. AppY also directly activates the expression of two operons involved in respiration but the consequences of these regulations on host physiology still have to be clarified. *appY* expression is induced by acid stress and probably by other environmental signals. AppY also leads to the stabilization of the sigma factor RpoS by an unknown mechanism. Under certain conditions, RpoS contributes to the expression of several genes under AppY control. The black arrows indicate the already known regulations, the blue arrows the regulations defined in this work and the dashed arrows regulations for which the molecular mechanism is not known.

In this paper, we characterized AppY, a transcriptional regulator whose gene is carried by the DLP12 prophage. After identifying AppY direct and indirect targets, we dissected the regulatory cascade leading to the modulation of three major processes, *i*.*e*. acid stress resistance, biofilm formation and motility, that were not linked to AppY before this study. Our results, summarized in Fig 7, provide evidence for how, at the molecular level, a prophage-encoded transcriptional regulator integrates into the host regulatory network at several entry points to drastically influence bacterial physiology.

A large number of the newly identified AppY-regulated genes are involved in the acid stress response (Figs 1–3, 5 and 7). Pathogenic as well as commensal *E*. *coli* strains have developed a great resistance to acid stress, certainly to cope with the acidic environment of the stomach in order to reach the mammalian gut. Five distinct acid resistant systems, named AR1 to AR5, contribute to acid resistance in *E*. *coli* [51–53]. These systems are not equally effective in dealing with acid stress; their efficacy varies depending on the medium composition and pH, as well as on the physiological state of the bacteria [31]. The majority of AppY regulated genes are part of the AR2 pathway and their expression is also regulated by one or more other regulators from the AraC/XylS family *i*.*e*. GadW, GadX and YdeO [31,34,35,40–42]. This apparent functional redundancy certainly hides a fine-tuned regulation where each of these regulators specifically responds to a defined stress by expressing the same set of genes. Such signal specificity has been previously shown for other regulators of the AraC/XylS family in the case of MarA, SoxS and Rob [54].

In addition to its role in acid stress tolerance, our results also identify a role for AppY in biofilm formation (Figs 4,5, 7 and S4 Fig). Biofilm formation is a complex process in which many cellular components are involved depending on the environmental conditions [55]. These cellular components include flagella, curli, exopolysaccharides and colonic acid. The increased biofilm formation observed when AppY is overproduced is due to the simultaneous and direct positive regulation of *nhaR* and *gadY* expression. The *nhaR* gene codes for a transcriptional regulator belonging to the LysR family and *gadY* for a small regulatory RNA. These two factors have been shown to regulate the *pgaABCD* operon which is responsible for the synthesis and export of the exopolysaccharide ß-1.6-poly-N glucosamine (PGA) involved in biofilm formation [38,39,56]. NhaR regulates this operon by directly binding to the *pgaABCD* promoter region, whereas GadY has been shown to titrate CsrA, a negative regulator of this operon. The concomitant activation of the AR2 system and of biofilm formation clearly shows that AppY activates different pathways to protect the cell from unfavorable environmental conditions.

AppY overproduction also massively represses the expression of genes coding for proteins involved in flagella synthesis leading to a drastic decrease in motility. This down-regulation is due, at least in part, to an active degradation of the master regulator of the flagella synthesis FlhDC by the HslUV protease (Figs 6 and 7). However, since AppY does not directly regulate the *hslUV* operon, an additional factor regulated by AppY and controlling *hslUV* expression remains to be identified. FlhDC degradation has already been observed in *Proteus mirabilis* and *Salmonella enterica* Typhimurium [49,50,57]. In these organisms the degradation depends on the Lon and ClpXP proteases, respectively. According to our data, AppY overproduction led to a mild increase in *lon* expression (2.5-fold) whereas *clpP* level remained constant in all tested conditions (S1 Table). This suggests that in *E*. *coli*, ClpXP does not play any role in FlhDC degradation whereas Lon may still contribute (S1 Table). This would be in accordance with the redundant role of HslUV and Lon already described for other substrates [58]. The regulatory cascades leading to FlhDC degradation in *P*. *mirabilis* and *S*. *enterica* Typhimurium have not yet been described. It would therefore be interesting to investigate if a protein from prophage origin could be involved as well in these two bacterial species. Indeed, the regulation of the master regulator FlhDC by a protein from prophage origin is not an isolated example. For instance, the Sp5 prophage inhibits the motility of the *E*. *coli* MG1655 strain by repressing *flhDC* expression, but also by decreasing FlhDC amount in the cell. To date, the protein encoded by Sp5 and responsible for this regulation has not been identified [59]. Motility regulation by morons is not restricted to swimming motility since twitching motility as well as swarming motility have all been described to be under the control of prophage genes [60–63]. This down-regulation of the flagella synthesis is consistent with biofilm formation and could also be a way to save energy under unfavorable growth conditions. This fits, once again, with an overall involvement of AppY in bacterial survival.

Prior to this study, the *app* and *hya* operons were the only characterized AppY targets [15,16,22–25]. Identifying these genes in the ChIP-Seq experiments both validates our approach and shows for the first time a direct AppY binding to the promoter regions of both operons (Figs 2A, 5C and 6). These two operons encode the cytochrome bd-II oxidase and the hydrogenase-I which are part of the respiratory machinery suggesting that AppY has also a direct role in regulating bacterial respiration. Interestingly, in addition to anaerobiosis, expression of the *hya* operon is also induced under acidic conditions (pH = 5.5) and this is partially dependent on AppY [29]. In another connection, a strain deleted for *cydB*, which codes for a subunit of the cytochrome bd-I oxidase, undergoes respiratory stress and displays a significant up-regulation of the *app* and *hya* operons as well as of the AR2 system and a down-regulation of flagellar genes [64]. The major overlap between these transcriptomic data and the AppY regulon described here suggests a strong interaction between respiratory stress and acid stress that probably needs to be investigated in more detail to fully understand AppY contribution to bacterial physiology.

Performing RNA-Seq experiments under AppY-overproducing conditions was a necessary step to fully appraise the AppY regulon. Indeed, although overproduction can favor unspecific DNA binding, it is also a convenient way to bypass the need for a potential signal or cofactor and to identify all the genes whose expression is controlled by a specific regulator, regardless of the conditions. Indeed, overproduction disrupts the equilibrium between the inactive and the active forms of transcriptional regulator by increasing the amount of active form, even when the activating signal is absent [65]. AppY has been shown to be active under several conditions such as carbon starvation, phosphate starvation, anaerobiosis or stationary phase [15,22]. However, in our experimental conditions, *appY* was not induced under anaerobiosis as previously described; the reason for this discrepancy is not known to date (S3A Fig). In this study, we identified low pH as an additional signal triggering *appY* expression (Fig 3B). It is now appealing to follow the dynamic of the AppY regulon under the different environmental conditions known to trigger *appY* expression. This will allow to determine if all AppY target genes are regulated under all conditions, if their expression varies depending on the encountered stress or if several simultaneous stresses are needed.

The binding site of most AraC/XylS family transcriptional regulators is 15 to 20 bp in length and overlaps with the -35 sequence of the promoter [66]. Under their dimeric form, these regulators may recognize pairs of binding sites in the same or inverted orientation, separated by a variable distance. However, a unique motif has already been described for transcriptional regulators of this family such as Ada or InvF [67,68]. In this study, we identified an 8-bp sequence overlapping the -35 region of *gadE* and *gadY* that, although probably not the entire motif recognized by AppY, contributes to AppY binding to DNA (Fig 5). Unless it is highly degenerated, no other motif can be predicted in the *gadY* promoter region; on the contrary, direct or inverted repeats can be predicted for *gadE*. Further *in vitro* experiments will be needed to identify the full AppY binding motif and to determine if AppY binds to this site as a dimer or as a monomer.

One striking observation from our ChIP-Seq data is that most of the AppY targets are also part of the RpoS regulon [69]. This raises the question of the functional link between these two regulators. Further experiments are needed to determine if RpoS and AppY work together or regulate the same pool of genes under different physiological conditions. RpoS is a sigma subunit of the RNA-polymerase allowing the transcription of an important number of genes [26]. When bacteria are growing in favorable conditions, RpoS is actively degraded by the ClpXP protease in the presence of the adaptor protein RssB [70–73]. Under stressful conditions however, different members of a family of proteins called Ira (Inhibitor of RssB activity) block RpoS degradation by sequestering RssB. So far, three Ira proteins have been identified in *E*. *coli*: IraP, IraD and IraM [27,74]. A previous study has shown that AppY also regulates this RpoS degradation pathway, independently of the known Ira proteins [27]. Our data confirm that AppY production does not up-regulate the expression of *iraP*, *iraD* or *iraM* (S1 Table). Since AppY regulates RpoS and RpoS has a huge impact on gene expression profiles, almost all the experiments presented here were performed in strains deleted of *rpoS*. Given the overlapping regulon between RpoS and AppY, these conditions were undoubtably necessary to unveil the dedicated AppY regulon. The stabilization of RpoS along with induction of the AR2 system suggest that AppY can induce both a specific and a general stress response. Several questions remain concerning the chronology of these responses: is there a hierarchy, with first a specific response followed by the general stress response if the applied stress persists? Does the activation of one or the other depend on different “state” of AppY or of the encountered stress? Do they occur simultaneously?

As already mentioned, AppY is encoded on the DLP12 prophage which is the most prevalent prophage in *Escherichia* genus and is also found in *Shigella* genomes [14]. DLP12 encodes also a second transcriptional regulator from the AraC/XylS, *ybcM* whose regulon has never been characterized. Interestingly, two other prophage-encoded transcriptional regulators from the AraC/XylS family, PsrB and PatE, also regulate genes from the AR2 system and enhance bacterial resistance to acidic conditions in the enterohemorrhagic *E*. *coli* O157:H7 strain [75,76]. This suggests that AppY is not an isolated case and that other prophage encoded members of the AraC/XylS family are integrated to the bacterial regulatory network to confer selective advantages to their host.

The work presented here highlights the significant impact that a prophage encoded regulator can have on its bacterial host. However, we can predict that it represents only a small portion of the huge regulatory network existing between ancestral bacterial genes and genes from prophages or, more widely, genes horizontally acquired. Consequently, studying the genetic interaction between phages and bacteria is key to fully understand bacterial physiology and how it influences bacterial evolution, environment and human health.

## Materials and methods

### Media and growth conditions

Cells were grown in Luria-Bertani (LB) broth. For acid stress experiments, we used E medium: (10 g/L MgSO_4_, 100 g/L citric acid, 500 g/L KH_2_PO_4_ and 175 g/L NaNH_4_HPO_4_ 4H_2_O), EG medium (E plus 0.4% D-Glucose) or potassium-modified LB (LBK; 10 g/L tryptone, 5 g/L of yeast extract, 7.45 g/L KCl) [77,78].

Liquid cultures were grown in aerobic conditions at 37°C under shaking (180 rpm) and plates were incubated at 37°C unless otherwise stated. The usual agar concentration for solid media is 1.5%. When required, antibiotics were added at the following concentrations: 100 μg/mL ampicillin (Amp), 25 μg/mL chloramphenicol (Cam), 50 μg/mL kanamycin (Kan), 10 μg/mL tetracycline (Tet) and 25 μg/mL zeocin (Zeo).

### Bacterial strains, plasmids and primers

Bacterial strains are listed in S2 Table, plasmids in S3 Table and primers in S4 Table. All strains are derivatives of *E*. *coli* str. K-12 substr. MG1655 constructed by P1 transduction, selected on the appropriate antibiotic and verified by PCR. To construct the *flhC*-SPA-*kan* strain (where SPA is a Sequential Peptide Affinity tag) or the deletion/insertion mutants of *gadE*, *gadY* and *nhaR*, we used the λ red recombination system [79,80] (see more details in S1 Methods). The P-*appY* translational fusion was constructed using the NM580 strain, which contains the mini-λ prophage and has been engineered to construct chromosomal *lacZ* fusions using a selection/counter-selection method [81] (see more details in S1 Methods). This fusion contains a fragment ranging from -615 bp to +24 bp relative to the ATG start codon of *appY* Open Reading Frame (ORF). Site-specific mutagenesis was performed to introduce the K170E mutation into pQE-AppY (primers BAΦ184/BAΦ185; codon AAA changed to AGA) [82]. Plasmids pND-671, pND-677 and pND-678 contain DNA fragments ranging from -598 pb to +14 pb relative to the *gadE* ATG start codon, from -245 bp to +14 bp relative to the beginning of the *gadY* sequence and from -210 bp to +15 bp relative to the ATG start codon for *gadA*. Plasmid pND-665 contains the full length *gadY* sequence and 245 nucleotides upstream. As we wanted to express *nhaR* from its own promoter region to keep the AppY-dependent induction and since it is in operon with *nhaA*, we amplified both genes plus a region of 482 nucleotides upstream *nhaA* ATG. PCR fragments were then digested with ClaI/BamHI for *gadY* and EcoRV/SalI for *nhaR* and cloned into pACYC184 digested with the same enzymes. The resulting plasmid is pND-692. Plasmid pND-807 contains *gadE* sequence plus 798 nucleotides upstream *gadE* ATG. All constructs were confirmed by Sanger sequencing.

### Chromatin immunoprecipitation sequencing (ChIP-Seq)

The ChIP-Seq protocol was adapted from [42,83]. ND3 strain transformed with the pQE80L empty vector or containing untagged *appY*, *appY-*3Flag or *appY*_K170E_-3Flag, was grown in LB/Amp medium to an OD_600_ of 0.2. Cultures were then induced with 0.05 mM IPTG and further grown for 1 h. This experiment was performed with 3 independent cultures (replicates R1, R2 and R3) for each condition. Cells were fixed with 1% formaldehyde; the crosslinking reaction was stopped by adding 250 mM glycine to the cultures. After being washed with PBS, the pellets were flash-frozen in liquid nitrogen and stored at -80°C. Cellular pellets were re-suspended in 1 mL of cold lysis buffer (50 mM Tris-HCl pH 7.5, 150 mM NaCl, 1 mM EDTA, 1% Triton-X100, protease inhibitor cocktail) plus 3.5 units of lysozyme (Novagen). Samples were then incubated at room temperature for 10 min on a rotating wheel (20 rpm) before shearing the crosslinked DNA by sonication at 4°C using a Bioruptor Standard Waterbath Sonicator (cycles of 30 sec ON/ 30 sec OFF, pulsing at maximum amplification for 10 min) (Diagenode). After centrifugation, 900 μL of the supernatant were mixed with 40 μL of Agarose Anti-Flag M2 gel beads (Sigma-Aldrich) and incubated overnight (ON) on a rotating wheel (20 rpm). After a gentle centrifugation step, the beads were washed sequentially with Low salt washing buffer, High salt washing buffer, LiCl washing buffer and twice with TBS buffer (see detailed composition in S1 Methods). Two elution steps of the Flag-tagged protein-DNA complexes were performed with 200 μL of Flag buffer (TBS buffer plus 100 μg/ml of 3Flag peptide from Sigma-Aldrich) and the samples were incubated 30 min at RT on a rotating wheel (20 rpm). After centrifugation, the protein-DNA crosslink was reversed by adding 25 μL of 5 M NaCl to the eluate and incubated ON at 65°C. Proteins and RNA were removed by adding sequentially proteinase K (NEB) and RNAse A (0.2 mg/ml) (Sigma). DNA was extracted with phenol/chloroform/isoamyl alcohol (25:24:1, Sigma-Aldrich) and ethanol precipitated. The DNA was finally resuspended in 30 μl of DNAse-free water, quantified with the Qubit dsDNA HS Assay kit (Invitrogen). DNA profiles were recorded with the TapeStation 4200 System (Agilent) in combination with the D5000 ScreenTape (Agilent). Libraries for high throughput DNA sequencing were prepared using the TruSeq ChIP Sample Preparation kit (Illumina) according to the manufacturer’s protocol starting with 10 ng dsDNA from the previous step. High throughput DNA sequencing was performed as described below.

### RNA sequencing (RNA-Seq)

The ND3 strain transformed with pQE80L or the pQE80L-*appY* was grown in LB/Amp medium to OD_600_ = 0.6 at 37°C under shaking (180 rpm). 1 mM IPTG was then added and cells grown for one additional hour. Total RNAs were then purified as described above. This experiment was performed with 3 independent cultures (replicates R1, R2 and R3) for each condition. Total RNAs samples (3 μg) were depleted from ribosomal RNA using the bacteria Ribo-Zero rRNA Removal kit (Illumina) according to the manufacturer’s protocol. The cDNA libraries were then prepared from the depleted RNA samples obtained during the previous step using the GoScript Reverse transcriptase (Promega) to synthesize the first strand cDNA and the TruSeq Stranded mRNA Library Prep kit (Illumina) according to the manufacturers’ protocols. High throughput DNA sequencing was performed as described below.

### RNA preparation and Reverse Transcription

For RNA-Seq and qRT-PCR experiments, 5.10^9^ cells were harvested and cellular pellets were flash-frozen in liquid nitrogen and stored at -80°C. Total RNAs were isolated from the pellet using the Maxwell 16 LEV miRNA Tissue Kit (Promega) according to the manufacturer’s instructions and an extra TURBO DNase (Invitrogen) digestion step to eliminate the contaminating DNA. The RNA quality was assessed by TapeStation 4200 system (Agilent). RNA was quantified spectrophotometrically at 260 nm (NanoDrop 1000; Thermo Fisher Scientific). For cDNA synthesis, 1 μg total RNA and 0.5 μg random primers (Promega) were used with the GoScript Reverse transcriptase (Promega) according to the manufacturer instructions.

### High throughput DNA sequencing

Prior to sequencing, RNA-Seq and ChIP-Seq libraries were quantified with the Qubit dsDNA HS Assay kit and their size distribution profiles recorded with the TapeStation 4200 System (Agilent) in combination with the D5000 DNA ScreenTape System (Agilent). Libraries were then diluted at 4 nM. Paired-end (2 x 75 bp) DNA sequencing was performed on the in-lab MiSeq sequencer hosted at the Institute Transcriptomic and Genomic facility with a MiSeq v3 (150-cycles) flow cell according to Illumina’s protocol. S5 Table summarizes the different sequencing runs performed during this study and the corresponding data yield for each sample.

Raw sequencing reads (FASTQ files trimmed from their Illumina adaptors) were submitted to the NCBI Sequence Read Archive under the BioProject accession number PRJNA751735.

### High throughput sequencing data analysis

The FASTQ files generated by the MiSeq sequencer were trimmed and clipped for quality control with Trimmomatic [84] using the following parameters: ILLUMINACLIP:TruSeq3-SE:2:30:10 LEADING:3 TRAILING:3 SLIDINGWINDOW:4:15 MINLEN:36. For subsequent analyses we retained only the paired-reads FASTQ files generated by Trimmomatics.

### RNA-Seq analysis

Trimmed paired-reads were mapped on *Escherichia coli* str. K12 substr. MG1655 reference genome (accession number U00096.3) and the raw read count per genomic feature computed with Rockhopper [85] using the following parameters: Orientation of mate-pair reads = rf, Max bases between paired reads = 500, Allowed mismatches = 0.15, Minimum seed length = 0.33, Min reads mapping to transcript = 10, Min transcript length = 50, Min count to seed a transcript = 50, Min count to extend a transcript = 5. Rockhopper mapping statistics are summarized in S6 Table. We observed fairly high variations in the efficiency of the ribo-depletion with as much as 36% of the reads mapping to rRNA in one sample. In order to obtain a proper data normalization in downstream analyses, we manually cured the read count matrix generated by Rockhopper from remaining rRNA read counts (16S-, 23S- and 5S- rRNA); the resulting trimmed matrix was then use as input for the differential gene expression analysis performed with Voom/Limma method. The latter is embedded in DEGUST (https://degust.erc.monash.edu/), an interactive web-tool for RNA-Seq analysis (https://doi.org/10.5281/zenodo.3258932), that allows to compare 3 different methods for differential gene expression analysis as well as easy data visualization, browsing and data export. All the RNA-Seq results are accessible on NCBI Sequence Read Archive under the BioProject accession number PRJNA751735. The RNA-Seq data are summarized in S1 Table.

### ChIP-Seq analysis

Illumina pair-end short reads were aligned on *Escherichia coli* str. K12 substr. MG1655 reference genome (accession number U00096.3) with Bowtie2 using default parameters [86]. The alignments were visualized and compared with IGV [87]. Detection and visualization of enriched loci along the genome were done with the SeqMonk software (https://www.bioinformatics.babraham.ac.uk/projects/seqmonk/). For DNA binding motive identification, BAM files generated by Bowtie2 were filtered to retain alignments with a minimum MAPQ quality of 20. Peak calling on the datasets obtained in the condition where 3Flag-tagged AppY is overproduced was then performed on a Galaxy platform (https://usegalaxy.org) with MACS (version 2.1.1.20160309) with the following parameters: Build model = create_model, Lower mfold bound = 3, Band width for picking regions to compute fragment size = 300, Peak detection based on qvalue, Minimum FDR (q-value) cutoff for peak detection = 0.05. Datasets obtained with the untagged AppY or the AppY_K170E_-3Flag overexpression served as ChIP-Seq control file. In order to predict an AppY DNA-binding sequence consensus, we used MEME and CentriMo from the MEME Suite [88]. As an input we used 201 bp DNA sequences centered on the absolute summit of each peak computed by MACS.

### Measure of expression with transcriptional *gfp* fusions

Strains ND3 or BAPHI089 were co-transformed with a plasmid carrying a *gfpmut2* transcriptional fusion and the pQE80L vector producing AppY wild-type (WT) or AppY_K170E_. ON cultures were diluted 60-fold into fresh LB medium supplemented with Amp, Kan and IPTG (0.05 mM) into a black 96-well plate with transparent bottom (Greiner). The plate was incubated into a Tecan Spark plate reader at 37°C with shaking (180 rpm) during 10 h. Every 10 min, the optical density at 600 nm (OD_600_) as well as the fluorescence intensity (excitation: 480 nm; emission: 535 nm) were measured. The transcriptional *gfp* fusion expression levels were standardized by dividing the fluorescence intensity by the OD_600_. Mean values and Standard deviation (SD) were computed on a minimum of three independent experiments.

### *appY* induction under acid conditions

This assay was carried out as previously described [43,44,77]. ON cultures of the BAPHI018 strain carrying the *appY* translational fusion were diluted 1:1000 in LBK medium buffered with 100 mM piperazine-N, N’-bis-2-(ethanesulfonic acid) and adjusted at pH 5.7 or 8.5 using KOH.

Anaerobic cultures were performed in screw-caped test tubes with a slow agitation on a rotating wheel (8 rpm); aerobic cultures were performed in 14 ml aerated polypropylene tubes in an orbital water bath (200 rpm). At OD_600_ = 0.4, samples were taken for β-galactosidase assay using the method described by Miller [89]. The pH of the cultures was verified at the end of the experiment to ensure that the values were maintained.

### Acid resistance assay

Acid resistance assays were performed as described previously [34]. Briefly, ON cultures were diluted 1:200 in 20 mL of LB at pH 7 with Amp and IPTG (1 mM) and grown at 37°C with aeration (180 rpm). At OD_600_ ≈ 1, 50 μL samples of culture were serially diluted in PBS and spotted on LB plates to count the colony forming cells (expressed in Colony Forming Unit or CFU). The resulting CFU represents the initial number of live cells before stress. In parallel, 50 μL of culture were transferred into 2 ml of preheated LB pH 2.5 (pH adjusted with HCl) and incubated for one hour at 37°C. 50 μL samples were serially diluted in PBS and 10 μL were spotted on LB plate. The CFU obtained here represents the cells having survived to the acid stress. The survival percentages were calculated by dividing the final CFU number by the initial CFU number. Each experiment was performed at least three times.

### Quantitative Real-Time-PCR for Transcriptional analysis

Quantitative real-time PCR (qPCR) and the corresponding analysis were performed on a CFX96 Real-Time System (Bio-Rad). The reaction volume was 15 μL and the final concentration of each primer was 0.5 μM. The cycling parameters of the qRT-PCR were 98°C for 2 min, followed by 45 cycles of 98°C for 5 s, 55°C for 10 s, 72°C for 1s. A final melting curve from 65°C to 95°C was performed to determine the specificity of the amplification. To determine the amplification kinetics of each product, the fluorescence derived from the incorporation of EvaGreen into the double-stranded PCR products was measured at the end of each cycle using the SsoFast EvaGreen Supermix 2X Kit (Bio-Rad, France). The results were analyzed using the Bio-Rad CFX Manager software, version 3.0 (Bio-Rad, France). The 16S RNA gene was used as a reference for normalization. For each point a technical duplicate was performed. The amplification efficiencies for each primer pairs were comprised between 80 and 100%. All of the primer pairs used for qRT-PCR are reported in the S4 Table.

### Pull down of AppY-3Flag with its DNA

Pull down we carried out using fluorescently Cy5-labbelled DNA fragments of *hyaA*, *gadE gadY* and *MalEK* promoter regions amplified by PCR. Fragments of 160 pb were amplified for *hyaA* and *gadE*, 200 pb for *gadY* and 442 pb for *MalEK*. Strain ND3 transformed with the pQE80L empty vector or containing *appY*-3Flag or *appY*_K170E_-3Flag was grown at 37°C in 100 ml LB medium plus Amp to an OD_600_ around 0.2. Cultures were then induced with 0.5 mM IPTG and grown for 1 hour. Cells were pelleted and store at -20°C. Pellets were resuspended in 4 ml of cold lysis buffer containing 50 mM Tris-HCL pH 7.5, 150 mM NaCl, 1 mM EDTA and protease inhibitor cocktail (Sigma). Samples were sonicated at 4°C using Branson Sonifier 450 (two cycles of 1 min with an output control of 2 and a duty control of 80%). After centrifugation, 600 μl of lysates were incubated with 50 μl of Agarose Anti-Flag M2 gel beads (Sigma-Aldrich) during 1 hour at 4°C on a rotating wheel to fix AppY-3Flag or AppY_K170E_-3Flag onto the beads. After a gentle centrifugation, beads were washed three times with 1 ml of lysis buffer. A mix containing 210 μl of Lysis buffer and 500 ng of target DNA tagged with Cy5 was made; 10 μl were kept as a loading control (input) and 100 μl were incubated with beads during 1 hour at room temperature on a rotating wheel. A sample of 10 μl of the supernatant was kept to observe the non-retained fraction (flowthrough). Input and flowthrough samples were loaded on a 1.5% TBE gel. Beads were washed three times. The elution step was done by incubating the beads with 20 μl of 2X loading buffer SDS at 25°C with agitation during 30 min. Eluates were loaded onto a gel 20% bis-acrylamide. The amount of eluted DNA, was detected by scanning gels with a FLA500 (Fuji) scanner (excitation wavelength: 635 nm (800 V scanning intensity) and emission wavelength: 665 nm). The amount of eluted protein was detected by western-blot using an anti-AppY antiserum.

### Motility assays

Motility assays were performed as described previously [34] on 0.5% Tryptone and 0.5% NaCl plates supplemented with 0.3% agar plus Amp, with or without 0.5 mM IPTG. ON cultures in LB plus Amp were standardized to an OD_600_ = 1 and 1 μl of each culture were spotted on plates. Plates were incubated at 30°C and after 15 to 20 h, the diameter of the swimming zone was measured using the ImageJ software [90]. All strains were tested in triplicate and each experiment was independently performed three time.

### Biofilm quantification assay

Biofilm quantification was performed according to [91]. ON cultures were diluted 1:1000 in fresh medium plus 0.5 mM IPTG in 24-well plate and incubated at 30°C without agitation for 24 h. Cultures were removed and the plate was washed two times with water before adding 0.1% crystal violet (Sigma-Aldrich) for 10 min. The plate was washed three times with water, let dry ON and samples were solubilized by using glacial acetic acid at 30% (Carlo Erba). After 15 min incubation, the OD_550_ was measured to quantify biofilm formation. Standard deviations are based on a minimum of three independent experiments.

### Protein electrophoresis and Western blotting

Samples were analyzed using 12% lab-made standard SDS-polyacrylamide gels for AppY detection and using 12% Precast Hepes-Tris gels (WSHT) for other proteins. Semidry electrophoretic transfer onto nitrocellulose membranes was performed using Trans-Blot Turbo Transfer System (BioRad). Membranes were probed with an anti-AppY antiserum (GenScript—4.4:10000 dilution), or a Flag antibody (SIGMA—1:10000 dilution) for FlhC-SPA detection and with HtpG antibody [92] (1:100000 dilution) as a loading control. The blots were developed with Immobilon Western (Millipore) using a chemiluminescence image analyzer (ImageQuant Las 4000, GE Healthcare).

### Statistical methods

Statistical analyses were done using the GraphPad Prism 8 software. Statistical significance calculations were analyzed using unpaired Student’s t test or one-way or two-way ANOVA with Tukey’s multiple comparisons test. P-values when differences are significant are provided on graphs and in figure legends.

## Supporting information

S1 Methods Supplemental materials and methods. (DOCX)

S1 Table Relative expression of genes under AppY overproduction (RNA-Seq data). Column 1: gene name. Column 2: Differential expression compared to the negative control (pQE80L empty vector). Columns 5 to 10: raw read counts per gene computed by Rockhopper. DS_pqe: MG1655 *rpoS*::*tet* transformed with pQE80L (empty vector). DS_AppY: MG1655 *rpoS*::*tet* transformed with pQE80L-AppY. R1, R2 and R3 stands for Replicate 1, 2 and 3, respectively.
(XLSX)

S2 Table DNA binding sites enrichment (AppY overproduction versus empty vector). Column 1: coordinate of the peak predicted by MACS (reference genome U00096.3). Columns 2–3: MACS prediction statistics. Column 4: name of the gene situated downstream the predicted peak (gene name synonym is given between brackets). When a peak falls in a region between two genes with no RNA-seq data available to hypothesize which gene is regulated, both gene names are noted, separated by "-". In green, chromosome loci chosen for DNA binding motif prediction by MEME suite.
(XLSX)

S3 Table Strains. (DOCX)

S4 Table Plasmids. (DOCX)

S5 Table Primers. (DOCX)

S6 Table RNA-Seq and ChIP-Seq sequencing data. (DOCX)

S7 Table Rockhopper mapping statistics. (DOCX)

S1 Fig Characterization of AppY-3Flag WT and K170E. A. Functionality of the different AppY constructs used in this study. pQE80L and pQE-*appY*_WT_ or pQE-*appY*_K170E_, with or without the 3-Flag tag were co-transformed in MG1655 Δ*rpoS* with the pUA66 empty vector (Cont) or the transcriptional fusions P_*appC*_*-gfp* and P_*hyaA*_*-gfp*. Cells were grown in LB at 37°C with 0.05 mM IPTG during 10 hours. Activity of the fusions was determined as described in Materials and Methods. The mean of 3 replicates is presented here and the standard deviation (SD) is indicated by the error bars. A.U., arbitrary units. Statistical significance calculations were performed using two-way ANOVA with Tukey’s multiple comparisons test (ns, not significant; ***, p-value < 0.001). B. Alignment of AppY C-terminal domain with other proteins from the AraC family. Sequence alignment was made using Jalview (12). AppY Helix-Turn-Helix motifs 1 and 2 are boxed in red. The intensity of the blue color reflects the residue conservation. The K170 residue mutated in this study is indicated by a red arrow. C. AppY_WT_ and AppY_K170E_ production. pQE80L empty vector (Cont) or containing *appY* (WT) or *appY* mutant (K170E) were transformed in MG1655 Δ*rpoS*. Cells were grown at 37°C in LB to OD_600_ ∼ 0.6 and 0.05 mM IPTG was added for 1 hour. AppY levels were analyzed by Western blotting using an anti-AppY antiserum.
(TIF)

S2 Fig AppY binding sites identified by ChIP-seq experiments. The genetic loci, the ChIP-Seq data obtained with AppY-3Flag and AppY_K170E_-3Flag and the gene expression profiles obtained by RNA-Seq with AppY or the vector control are shown from the top to the bottom. Peaks observed in ChIP-Seq with AppY_WT_ (blue) or AppY_K170E_ (black) are superimposed for comparison; the intensity of the red color in RNA-Seq panels represents the number of raw counts per gene. The data are representative of three independent experiments.
(TIF)

S3 Fig AppY contribution to acid stress. A. Strains carrying a chromosomal *appY-lacZ* translational fusion were cultured ON in LBK pH = 7, diluted 1:1000 into LB pH = 8.5 (dotted line) or 5.7 (plain) and incubated at 37°C in aerobic (light blue) or anaerobic (dark blue) conditions. Cultures were grown until an OD_600_ ∼ 0.4. The activity of the *appY* fusion was determined as described using the Miller assay (13). Data are means +/- standard deviation (n = 3). Statistical significance calculations were performed using one-way ANOVA with Tukey’s multiple comparisons test (ns, not significant; *, p-value < 0.05; **, p-value < 0.01). B. Experiments were performed as described in A with a WT (blue) and Δ*evgA* (grey) strains in aerobic conditions C. Strains BW25113 (WT) and Δ*gadC* or MG1655 (WT) and Δ*appY*, were grown in LB plus 0.4% glucose at 37°C for 22 hours. Cultures were diluted 1:1000 into EG medium pH = 2.2 and grown with or without sodium glutamate for 4 hours. Cells were serially diluted and 10 𝜇l of cultures were spotted on LB plate incubated at 37°C. D. AppY overproduction confers resistance to acid stress. MG1655 WT, Δ*rpoS*, Δ*gadE* or Δ*gadE* Δ*rpoS* strains transformed with pQE80L (-), pQE-*appY*_WT_ (WT) or pQE-*appY*_K170E_ (K170E) were grown to OD_600_ = 1 in LB broth (pH 7.0) with 1 mM IPTG. Cells were diluted 40-fold into LB broth (pH 2.5) and incubated for 1 h at 37°C. Cells were serially diluted and 10 μl of cultures were spotted on LB plate incubated at 37°C. E. *gadE* expressed under its own promotor restores cell survival when AppY is overproduced. Strains MG1655 and *ΔgadE* were co-transformed with pQE80L (-) or pQE-*appY*_WT_ (WT) and pACYC184 (-) or pACYC184-*gadE*. The experiment was carried out as described in S3.D.
(TIF)

S4 Fig AppY favors biofilm formation *via* the direct induction of *nhaR* and *gadY*. The indicated strains were grown in LB plus 0.5 mM IPTG at 30°C without shaking for 24 hours. Biofilm was visualized using crystal violet staining. A. Biofilm formation dependent on NhaR and GadY. MG1655 Δ*rpoS*, MG1655 Δ*rpoS* Δ*gadY* or MG1655 Δ*rpoS* Δ*nhaR* were transformed with the pQE80L empty vector (-) or containing *appY* (WT) or *appY* mutant (K170E). B. Complementation of MG1655 Δ*rpoS* Δ*gadY* with *gadY and nhaR* expressed under their own promoter. MG1655 Δ*rpoS* Δ*gadY* strain was co-transformed with a pQE80L construct (empty vector (-), containing *appY* (WT) or *appY* mutant (K170E)) and a pACYC184 construct (empty vector (p-Cont), containing *nhaR* (p-*nhaR*) or *gadY* (p-*gadY*)). C. Complementation of MG1655 Δ*rpoS* Δ*nhaR* with *gadY and nhaR* expressed under their own promoter. MG1655 Δ*rpoS* Δ*nhaR* strain was co-transformed with a pQE80L construct (empty vector (-), containing *appY* (WT) or *appY* mutant (K170E)) and a pACYC184 construct (empty vector (p-Cont), containing *nhaR* (p-*nhaR*) or *gadY* (p-*gadY*)).
(TIF)

S5 Fig Input and flowthrough of pull-down assay. A solution of lysis buffer containing 500 ng of DNA was equally distributed in tubes containing the beads previously incubated with AppY-3Flag or AppY_K170E_-3Flag. Unspecific DNA binding to the beads, was checked by pooling all the DNA fragments and incubate them with the beads treated with a lysate containing only the pQE empty vector. To estimate the initial amount of DNA in our samples, 10 μl was loaded on a 1.5% TBE gel (Input). After DNA incubation with the beads, 10 μl of flowthrough (FT) was loaded on the same gel to estimate the amount of unbound DNA. Gel was scanned using a FLA500 (Fuji) scanner (excitation wavelength: 635 nm (800 V scanning intensity); emission wavelength: 665).
(TIF)
