# Supplementary material for: The prophage-encoded transcriptional regulator AppY has pleiotropic effects on E. coli physiology
Source: PLoS Genet. 2023 Mar 17;19(3):e1010672. doi: 10.1371/journal.pgen.1010672 (PMC10057817; doi:10.1371/journal.pgen.1010672)
Supplement: S1 Methods — (DOCX) [file pgen.1010672.s001.docx]

**SUPPORTING INFORMATION:**

**Supplemental Materials and Methods**

**Supplemental Tables:**

**S1 Table: Relative expression of genes under AppY overproduction (RNA-Seq data)**

**S2 Table: DNA binding sites enrichment (AppY overproduction versus empty vector)**

**S3 Table: Strains**

**S4 Table: Plasmids**

**S5 Table: Primers**

**S6 Table: RNA-Seq and ChIP-Seq sequencing data**

**S7 Table: Rockhopper mapping statistics**

**Supplemental Figures:**

**S1 Figure: Characterization of AppY-3Flag WT and K170E**

**S2 Figure: AppY binding sites identified by ChIP-seq experiments**

**S3 Figure: AppY contribution to acid stress**

**S4 Figure: AppY favors biofilm formation *via* the direct induction of *nhaR* and *gadY*.**

**S5 Figure: Input and Flowthrough of pull-down assay.**

**Supplemental Materials and Methods:**

**Bacterial strains, plasmids and primers**

To construct the *flhC*-SPA-*kan* strain or the deletion/insertion mutants of *gadY*, *nhaR* and *gadE* we used the λ Red recombination system. Briefly, the SPA-*kan*, the kanamycin resistance and the chloramphenicol resistance cassettes were amplified using as a template the BAPHI010 strain (primers BAΦ510/511), the BAPHI046 strain (primers BAΦ246/BAΦ247 for *gadY* and BAΦ359/BAΦ360 for *nhaR*) and the TKC strain (primers BAΦ084/BAΦ085), respectively (1–3). Primers used are 60 nucleotides long, they contain 20 nucleotides homologous to the desired cassette and 40 nucleotides homologous to the region flanking the coding sequences of *gadE*, *gadY* and *nhaR* for mutant’s construction; for *flhC*-SPA-*kan* homology has to be on both sides of the STOP codon*.* The resulting PCR products were transformed into the NM1100 strain containing a mini-λ prophage and recombined into the bacterial chromosome thanks to the λ-red functions (4,5). Transformed cells were selected on the appropriate antibiotic and checked by PCR.

To construct the P-*appY* translational fusion, the primers used contains 40 nucleotides homologous to the region of insertion in NM580, corresponding to the zeocin cassette on the 5’end and to the *lacZ* ORF starting at the 9^th^ codon on the 3’ end. After transformation, the cells were recovered overnight on the bench in LB containing 1% glucose and plated on LB 1% arabinose to favor counter-selection. The resulting clones were screened for Kan^S^, checked by PCR and sequenced. In the ND79 and ND85 strains, the gene conferring resistance to kanamycin was removed using pCP20 (6) to allow the transduction of other *kan* containing alleles.

**Chromatin Immunoprecipitation Sequencing (ChIP-Seq)**

Composition of buffers used for ChIP-Seq:

- Low salt washing buffer (0.1 % SDS, 1 % Triton-X100, 2 mM EDTA, 20 mM Tris-HCl pH 8.1, 150 mM NaCl)

- High salt washing buffer (0.1% SDS, 1% Triton-X100, 2 mM EDTA, 20 mM Tris-HCl pH 8.1, 500 mM NaCl)

- LiCl washing buffer (0.25 M LiCl, 1 % IGEPAL®, 1 % sodium deoxycholate, 1 mM EDTA, 10 mM Tris-HCl pH 8.1)

- TBS buffer (50 mM Tris-HCl pH 7.5, 150 mM NaCl).

**Gene ontology classification**

For an overview of the RNA-seq experiment, we grouped the highly differentially expressed genes (|fold-change| ≥ 10) according to their biological processes. Based on Gene Ontology (GO) numbers gathered from Ecocyc (PMID: 34394059), we defined 11 functional families : bacterial motility (GO:0071973, GO:0071978, GO:0071977, GO:1902201), biofilm (GO:0043709, GO:1900232), catabolism (GO:0016052, GO:0006543, GO:0030163, GO: 0006212, GO:0046397), lipopolysaccharide metabolism (GO:0008653, GO:009103), protein maturation (GO:0016485, GO:0051604), anaerobic or aerobic respiration (GO:0071454, GO:0019646, GO:0019645), response to pH stress (GO:0051454, GO:1990451, GO:0010447, GO:0009268, GO:0071468), response to osmotic stress (GO:0006972, GO:0006970), transcription regulation (GO:0045892, GO:0045893), membrane transport (GO:0006865, GO:0042908, GO:0015912, GO:0042953, GO:0055085, GO:0071577) and others.

**Acid stress assay specific to the AR2 system**

This assay was performed according to (7). Strains were grown for 22 hours at 37°C with aeration in LB medium with 0.4 % glucose at pH 7. These cultures were then diluted 1:1000 in 5 mL of EG medium at pH 2.2 (pH adjusted with HCl) supplemented with 1.5 mM sodium glutamate. As a control of the AR2 system induction, we used a medium corresponding to the EG medium at pH 2.2 without any additives. 100 µL samples of each culture were collected after 0, 2 and 4 hours of aerobic growth at 37°C. These samples were serially diluted in EG medium at pH 7, 10 µL were spotted on LB plate and incubated at 37°C.

**REFERENCES**

1. Datsenko KA, Wanner BL. One-step inactivation of chromosomal genes in Escherichia coli K-12 using PCR products. Proceedings of the National Academy of Sciences. 6 juin 2000;97(12):6640‑5.

2. Sharan SK, Thomason LC, Kuznetsov SG, Court DL. Recombineering: A Homologous Recombination-Based Method of Genetic Engineering. Nat Protoc. 2009;4(2):206‑23.

3. Zeghouf M, Li J, Butland G, Borkowska A, Canadien V, Richards D, et al. Sequential Peptide Affinity (SPA) System for the Identification of Mammalian and Bacterial Protein Complexes. J Proteome Res. juin 2004;3(3):463‑8.

4. Bougdour A, Cunning C, Baptiste PJ, Elliott T, Gottesman S. Multiple pathways for regulation of sigmaS (RpoS) stability in Escherichia coli via the action of multiple anti-adaptors. Mol Microbiol. avr 2008;68(2):298‑313.

5. Court DL, Swaminathan S, Yu D, Wilson H, Baker T, Bubunenko M, et al. Mini-λ: a tractable system for chromosome and BAC engineering. Gene. oct 2003;315:63‑9.

6. Cherepanov PP, Wackernagel W. Gene disruption in Escherichia coli: TcR and KmR cassettes with the option of Flp-catalyzed excision of the antibiotic-resistance determinant. Gene. janv 1995;158(1):9‑14.

7. Castanie-Cornet MP, Penfound TA, Smith D, Elliott JF, Foster JW. Control of Acid Resistance in *Escherichia coli*. J Bacteriol. juin 1999;181(11):3525‑35.
