# Supplementary material for: The prophage-encoded transcriptional regulator AppY has pleiotropic effects on E. coli physiology
Source: PLoS Genet. 2023 Mar 17;19(3):e1010672. doi: 10.1371/journal.pgen.1010672 (PMC10057817; doi:10.1371/journal.pgen.1010672)
Supplement: S3 Table — (DOCX) [file pgen.1010672.s004.docx]

S3 Table: Strains

| **Strain** | **Description** | **Reference or source** |
| --- | --- | --- |
| MG1655 | *E. coli* K12 substr. MG1655 | Acc. number U00096.3 |
| BW25113 | *lacI+ rrnB_T14_ ΔlacZ_WJ16_ hsdR514 ΔaraBAD_AH33_ ΔrhaBAD_LD78_ rph-1 Δ(araB–D)567 Δ(rhaD-B)568 ΔlacZ4787(::rrnB-3) hsdR514 rph-1* | [1] |
| BA367 | MG1655 *crl-* *rpoS::tet* | Gottesman S., lab collection |
| C600 | *F- e14^-^ (Mcr^-^) or e14^-^ (McrA^+^) thr-1 leuB6 thi-1 lacY1 supE44 rfbD1 fhuA25* | [2] |
| NM580 | MG1655 *lacI-T1T2-zeo^R^-pBRpLacO-kan-pBAD-ccdB. Mini-λ-Red::tet^R^* | [3] |
| NM1100 | *Mini-λ-Red::tet^R^* | [4] |
| SG22098 | MC4100 *clpP::cat* | [5] |
| TKC | *tet*, *kan*, *cat* containing *E. coli* strain | [6] |
| BAPHI010 | NM1100 AppY-SPA | NM1100 + PCR (BAΦ004/BAΦ005) |
| BAPHI018 | MG1655 *zeo^R^-PappY-*SD-ATG + 8 codons *appY-* 9^th^ codon *lacZ* | NM580 + PCR (BAΦ001/BAΦ002) |
| BAPHI046 | MG1655 *lon::kan* | MG1655 + P1 (JW0429) |
| BAPHI056 | MG1655 *appY::kan* | MG1655 + P1 (JW0553) |
| BAPHI089 | MG1655 *rpos::tet gadE::cat* | ND3 + P1 (ND34) |
| ND2 | MG1655 *appY::kan rpoS::tet* | BAPHI056 + P1 (BA367) |
| ND3 | MG1655 *rpoS::tet* | MG1655 + P1 (BA367) |
| ND17 | MG1655 *gadE::cat* | MG1655 + P1 (ND34) |
| ND34 | NM1100 *gadE::cat* | NM1100 + PCR (BAΦ084/BAΦ085) |
| ND50 | MG1655 *gadY::kan* | NM1100 + PCR (BAΦ246/BAΦ247) |
| ND51 | MG1655 *rpoS::tet gadY::kan* | ND3 + P1 (ND50) |
| ND55 | MG1655 *nhaR::kan* | NM1100 + PCR (BAΦ359/BAΦ360) |
| ND56 | MG1655 *rpoS::tet nhaR::kan* | ND3 + P1 (ND55) |
| ND74 | MG1655 *rpoS::tet flhC-SPA-kan* | ND3 + P1 (*flhC*-SPA-*kan*) |
| ND76 | MG1655 *rpoS::tet clpP::cat flhC-SPA-kan* | ND78 + P1 (*flhC*-SPA-*kan*) |
| ND77 | MG1655 *zeo^R^-PappY-*SD-ATG + 8 codons *appY-* 9^th^ codon *lacZ, evgA::kan* | BAPHI018 + P1 (JW2366) |
| ND78 | MG1655 *rpoS::tet clpP::cat.* | ND3 + P1 (SG22098) |
| ND79 | MG1655 *Δlon°* | BAPHI046 + pCP20 |
| ND80 | MG1655 *Δlon° rpoS::tet* | ND79 + P1 (BA367) |
| ND82 | MG1655 *Δlon° rpoS::tet flhC-*SPA° | ND80 + P1 (*flhC*-SPA-*kan*) |
| ND85 | MG1655 *rpoS::tet flhC-*SPA° | ND74 + pCP20 |
| ND86 | MG1655 *rpoS::tet* *hslU::kan*  *flhC-*SPA° | ND85 + P1 (JW3902) |
| JW0429 | BW25113 *lon::kan* | [7] |
| JW0553 | BW25113 *appY::kan* | [7] |
| JW1487 | BW25113 *gadC::kan* | [7] |
| JW2366 | BW25113 *evgA::kan* | [7] |
| JW3902 | BW25113 *hslU::kan* | [7] |
| JW3480 | BW25113 *gadE::kan* | [7] |
| ND112 | MG1655 *gadE::kan* | MG1655 + P1 (JW3480) |

**REFERENCES**

1. Datsenko KA, Wanner BL. One-step inactivation of chromosomal genes in Escherichia coli K-12 using PCR products. Proceedings of the National Academy of Sciences. 2000;97: 6640–6645. doi:10.1073/pnas.120163297

2. Bachmann 1996. In: Neidhardt et al., eds. ASM:2460-2488. [cited 28 Feb 2022]. Available: https://cgsc.biology.yale.edu/Reference.php?ID=41187

3. Battesti A, Majdalani N, Gottesman S. Stress sigma factor RpoS degradation and translation are sensitive to the state of central metabolism. Proc Natl Acad Sci USA. 2015;112: 5159–5164. doi:10.1073/pnas.1504639112

4. Bougdour A, Cunning C, Baptiste PJ, Elliott T, Gottesman S. Multiple pathways for regulation of sigmaS (RpoS) stability in Escherichia coli via the action of multiple anti-adaptors. Mol Microbiol. 2008;68: 298–313. doi:10.1111/j.1365-2958.2008.06146.x

5. Lehnherr H, Yarmolinsky MB. Addiction protein Phd of plasmid prophage P1 is a substrate of the ClpXP serine protease of Escherichia coli. PNAS. 1995;92: 3274–3277. doi:10.1073/pnas.92.8.3274

6. Sharan SK, Thomason LC, Kuznetsov SG, Court DL. Recombineering: A Homologous Recombination-Based Method of Genetic Engineering. Nat Protoc. 2009;4: 206–223. doi:10.1038/nprot.2008.227

7. Baba T, Ara T, Hasegawa M, Takai Y, Okumura Y, Baba M, et al. Construction of Escherichia coli K-12 in-frame, single-gene knockout mutants: the Keio collection. MolSystBiol. 2006;2: 2006.
