# Supplementary material for: The prophage-encoded transcriptional regulator AppY has pleiotropic effects on E. coli physiology
Source: PLoS Genet. 2023 Mar 17;19(3):e1010672. doi: 10.1371/journal.pgen.1010672 (PMC10057817; doi:10.1371/journal.pgen.1010672)
Supplement: S4 Table — (DOCX) [file pgen.1010672.s005.docx]

S4 Table: Plasmids

| Plasmid | Description | Reference |
| --- | --- | --- |
|  | pQE80L, Amp^R^, ColE1 replication origin, T5 promoter | Qiagen |
|  | pQE80L-AppY, Amp^R^ | [1] |
| pND-572 | pQE80L-AppY_K170E_, Amp^R^ | Directed mutagenesis on pQE80L-AppY with BAΦ184/BAΦ185 |
| pND-574 | pQE80L-AppY-3Flag, Amp^R^ | PCR BAΦ075/BAΦ173, digested EcoRI/HindIII and inserted into pQE80L |
| pND-610 | pQE80L-AppY_K170E_-3Flag, Amp^R^ | Directed mutagenesis on pND574 with BAΦ184/BAΦ185 |
|  | pUA66, Km^R^, sc101 replication origin | [2] |
|  | pUA66-PappC, Kan^R^ | [2] |
|  | pUA66-P*gadB*, Kan^R^ | [2] |
|  | pUA66-P*gadX*, Kan^R^ | [2] |
|  | pUA66-P*hdeA*, Kan^R^ | [2] |
|  | pUA66-P*hdeD*, Kan^R^ | [2] |
|  | pUA66-P*hyaA*, Kan^R^ | [2] |
|  | pUA66-P*slp*, Kan^R^ | [2] |
| pND-671 | pUA66-P*gadE*, Kan^R^ | PCR with BAΦ227/BAΦ228, digested XhoI/BamHI inserted into pUA66 |
| pND-797 | pUA66-P*gadE_gcaa_ ,* Kan^R^ | Directed mutagenesis on pND-671 with BAΦ769/BAΦ770 |
| pND-677 | pUA66-P*gadY*, Kan^R^ | PCR with BAΦ236/BAΦ237, digested XhoI/BamHI inserted into pUA66 |
| pND-805 | pUA66-P*gadY_gcaa_*, Kan^R^ | Directed mutagenesis on pND-677 with BAΦ773/BAΦ774 |
| pND-678 | pUA66-P*gadA*, Kan^R^ | PCR with BAΦ238/BAΦ239, digested XhoI/BamHI inserted into pUA66 |
|  | pACYC184, Cam^R^, p15A replication origin | Biolabs |
| pND-665 | pACYC184-*gadY*, Cam^R^ | PCR with BAΦ371/BAΦ372, digested ClaI/BamHI and introduced into pACYC184 |
| pND-692 | pACYC184-*nhaA-nhaR*, Cam^R^ | PCR with BAΦ584/BAΦ586, digested EcoRV/SalI and introduced into pACYC184 |
| pND-807 | pACYC184-*gadE,* Cam^R^ | PCR with BAΦ812/ BAΦ813 digested EcoRV/BamHI and introduced into pACYC184 |

**REFERENCES**

1. Bougdour A, Cunning C, Baptiste PJ, Elliott T, Gottesman S. Multiple pathways for regulation of sigmaS (RpoS) stability in Escherichia coli via the action of multiple anti-adaptors. Mol Microbiol. 2008;68: 298–313. doi:10.1111/j.1365-2958.2008.06146.x

2. Zaslaver A, Bren A, Ronen M, Itzkovitz S, Kikoin I, Shavit S, et al. A comprehensive library of fluorescent transcriptional reporters for Escherichia coli. Nat Methods. 2006;3: 623–628. doi:10.1038/nmeth895
