# Supplementary material for: The prophage-encoded transcriptional regulator AppY has pleiotropic effects on E. coli physiology
Source: PLoS Genet. 2023 Mar 17;19(3):e1010672. doi: 10.1371/journal.pgen.1010672 (PMC10057817; doi:10.1371/journal.pgen.1010672)
Supplement: S5 Table — (DOCX) [file pgen.1010672.s006.docx]

S5 Table: Primers

| Primer number | Primer name | Primer sequence (5’-3’) |
| --- | --- | --- |
| BAФ001 | Chromosomal *appY* translational *lacZ* fusion Fwd | TAC-TAT-GCC-GAT-ATA-CTA-TGC-CGA-TGA-TTA-ATT-GTC-AAC-GTA-TCG-GGT-GCT-GCT-AAA-CC |
| BAФ002 | Chromosomal *appY* translational *lacZ* fusion Rev | CCA-GGG-TTT-TCC-CAG-TCA-CGA-CGT-TGT-AAA-ACG-ACG-GCA-ACT-ACG-GAG-CAA-ACA-TAA-TC |
| BAФ004 | Chromosomal *appY*-SPA Fwd | AAA-ATA-ATC-GGC-GTC-ACA-GAT-GGA-ATA-AAC-AAA-ACA-ATT-GAC-TCC-ATG-GAA-AAG-AGA-A |
| BAФ005 | Chromosomal *appY*-SPA Rev | TAT-TAT-AAT-TAA-CAT-GTA-GAC-AAC-TTG-TAA-TAA-ACA-TTA-CAT-ATG-AAT-ATC-CTC-CTT-AG |
| BAФ075 | *appY*-3Flag Fwd | ATC-GGA-ATT-CAT-TAA-AGA-GGA-GAA-ATT-AAC-TAT-GGA-TTA-TGT-TTG-CTC-CGT-AGT-TTT-CAT |
| BAФ084 | *gadE::cam* Fwd | GGA-TGA-CAT-ATT-CGA-AAC-GAT-AAC-GGC-TAA-GGA-GCA-AGT-TTG-TGA-CGG-AAG-ATC-ACT-TCG |
| BAФ085 | *gadE::cam* Rev | CTC-GTC-ATG-CCA-GCC-ATC-AAT-TTC-AGT-TGC-TTA-TGT-CCT-GAA-CCA-GCA-ATA-GAC-ATA-AGC-G |
| BAФ147 | qRT-PCR, *appC* Fwd | GAT-GGA-AGG-GGA-GTG-GCA-AA |
| BAФ148 | qRT-PCR, *appC* Rev | CGT-GGG-TAG-GTT-TCA-GCC-AT |
| BAФ149 | qRT-PCR, *fliA* Fwd | AGC-GAG-AAA-ACC-CGC-TAC-AA |
| BAФ150 | qRT-PCR, *fliA* Rev | TGT-GTA-ACT-GAC-TGA-CCC-GC |
| BAФ151 | qRT-PCR, *flgB* Fwd | CAA-TGC-CGA-TAC-CCC-TGG-TT |
| BAФ152 | qRT-PCR, *flgB* Rev | TTG-CAG-TTC-TGC-GGT-AGG-AG |
| BAФ153 | qRT-PCR, *fliF* Fwd | CTG-CGT-GCG-AAT-CCG-AAA-AT |
| BAФ154 | qRT-PCR, *fliF* Rev | CTT-CGC-TGA-AGC-GGT-AAG-GA |
| BAФ155 | qRT-PCR, *fliL* Fwd | TTT-GCC-GGA-AGT-CCG-TAG-TC |
| BAФ156 | qRT-PCR, *fliL* Rev | TCG-GTG-ACA-TCC-TGT-TTC-GG |
| BAФ173 | *appY*-3Flag Rev | GAT-CAA-GCT-TCT-ACT-TGT-CAT-CGT-CAT-CCT-TGT-AGT-CGA-TGT-CAT-GAT-CTT-TAT-AAT-CAC-CGT-CAT-GGT-CTT-TGT-AGT-CGT-CAA-TTG-TTT-TGT-TTA-TTC |
| BAФ184 | AppY K170E Fwd | GAA-AGT-TTA-ATA-GAA-AAA-AGA-TTA-A |
| BAФ185 | AppY K170E Rev | TTA-ATC-TTT-TTT-CTA-TTA-AAC-TTT-C |
| BAФ227 | pUA66-*gadE* Fwd | GAT-GCT-CGA-GTT-ACC-CCG-GTT-GTC-ACC-CGG |
| BAФ228 | pUA66-*gadE* Rev | GAT-CGG-ATC-CGT-CAT-GAG-AAA-AAT-CAT-AAC |
| BAФ236 | pUA66-*gadY* Fwd | GAT-GCT-CGA-GGA-TTA-TCC-CTT-ATA-TTT-CAT-AC |
| BAФ237 | pUA66-*gadY* Rev | GAT-CGG-ATC-CAA-CTT-TGT-GCT-CTC-AGT-AAG |
| BAФ238 | pUA66-*gadA* Fwd | GAT-GCT-CGA-GTT-AAT-TTG-ATC-GCC-CGA-ACA-G |
| BAФ239 | pUA66-*gadA* Rev | GAT-CGG-ATC-CAA-CAG-CTT-CTG-GTC-CAT-TTC |
| BAФ246 | *gadY::k*an Fwd | AAT-GGC-TGA-TCT-TAT-TTC-CAG-TAA-AAG-TTA-TAT-TTA-ACT-TAT-TCC-GGG-GAT-CCG-TCG-ACC |
| BAФ247 | *gadY::k*an Rev | CTG-CGG-AAG-GAA-TAA-GAT-TAT-AGA-GTT-TTA-CTC-AGA-CAT-ATG-TAG-GCT-GGA-GCT-GCT-TCG |
| BAФ359 | *nhaR::k*an Fwd | GCC-ATA-AAC-GGC-TCC-CTT-TTC-ATT-GTT-ATC-AGG-GAG-AGA-AAT-TCC-GGG-GAT-CCG-TCG-ACC |
| BAФ360 | *nhaR::k*an Rev | CGC-ACC-GCT-GGA-CTA-AAA-AGC-GCA-GAA-TAA-TCC-GTA-TTG-CTG-TAG-GCT-GGA-GCT-GCT-TCG |
| BAФ371 | pACYC184-*gadY* Fwd | CTA-GAT-CGA-TGA-TTA-TCC-CTT-ATA-TTT-CAT-AC |
| BAФ372 | pACYC184-*gadY R*ev | GAT-CGG-ATC-CAA-AAA-AAC-CCG-GCA-TAG-GGG |
| BAФ376 | qRT-PCR, *flhD* Fwd | TCC-GCT-ATG-TTT-CGT-CTC-GG |
| BAФ377 | qRT-PCR, *flhD* Rev | ATC-GTC-AAC-GCG-GGA-ATC-TT |
| BAФ510 | Chromosomal *flhC-*SPA Fwd | TAT-CCC-ACA-ACT-GCT-GGA-TGA-ACA-GAG-AGT-ACA-GGC-TGT-TTC-CAT-GGA-AAA-GAG-AAG |
| BAФ511 | Chromosomal *flhC-*SPA Rev | GTC-GTT-ACC-GCT-GCT-GGA-ATG-TTG-CGC-CTC-ACC-GTA-TCA-GCA-TAT-GAA-TAT-CCT-CCT-TAG |
| BAФ584 | pACYC-*nhaA*-*nhaR* Fwd | CTA-GGA-TAT-CCT-ATC-TGC-CGT-TCA-GCT-AAT-G |
| BAФ586 | pACYC-*nhaA*-*nhaR* Rev | GAT-CGT-CGA-CTT-AAC-GCA-CCG-CTG-GAC-TAA-AAA-G |
| BAФ769 | pUA66-P*gadE_gcaa_* Fwd | TTC-AAA-CAT-TAT-CGC-AAC-TGA-TAT-TTT-CCG-TA |
| BAФ770 | pUA66-P*gadE_gcaa_* Rev | TAC-GGA-AAA-TAT-CAG-TTG-CGA-TAA-TGT-TTG-AA |
| BAФ773 | pUA66-P*gadY_gcaa_* Fwd | TTA-TAA-AAA-AGC-AAC-TGA-TCT-TAT-TTC-CAG-TA |
| BAФ774 | pUA66-P*gadY_gcaa_* Rev | TAC-TGG-AAA-TAA-GAT-CAG-TTG-CTT-TTT-TAT-AA |
| BAФ812 | pACYC-*gadE* Fwd | GAT-CGA-TAT-CTT-ACC-CCG-GTT-GTC-ACC-CGG-A |
| BAФ813 | pACYC-*gadE* Rev | GAT-CGG-ATC-CCT-AAA-AAT-AAG-ATG-TGA-TAC-C |
| BAФ498 | Promoter region *gadE* Fwd | ACC-TGC-TAG-TGA-TTA-TTT-CA |
| BAФ777 | Promoter region *gadE* Rev | AAC-GCT-TAC-GGA-ACT-CTT-TGT |
| BAФ500 | Promoter region *gadY* Fwd | ATT-TGG-TAG-GAC-CAA-AAT-TTA |
| BAФ778 | Promoter region *gadY* Rev | CCG-TTA-TAA-CAC-TCC-CTG-TTG |
| BAФ504 | Promoter region *hyaA* Fwd | GCT-AAA-CAT-CCC-CAC-CGC-CC |
| BAФ779 | Promoter region *hyaA* Rev | ACG-AAA-GCT-CAT-CCT-TCT-GCA |
|  | Promoter region *malEK* Fwd | ATG-CGA-GGA-TGC-GTG-CAC-CT |
|  | Promoter region *malEK* Rev | CTC-GCC-CCA-GGC-TTT-CGT-TA |
| 16S-EC1 | qRT-PCR, 16S RNA Fwd | GTT-AAT-ACC-TTT-GCT-CAT-TGA |
| 16S-EC2 | qRT-PCR, 16S RNA Rev | ACC-AGG-GTA-TCT-AAT-CCT-GTT |
