# Supplementary material for: The prophage-encoded transcriptional regulator AppY has pleiotropic effects on E. coli physiology
Source: PLoS Genet. 2023 Mar 17;19(3):e1010672. doi: 10.1371/journal.pgen.1010672 (PMC10057817; doi:10.1371/journal.pgen.1010672)
Supplement: S6 Table — (DOCX) [file pgen.1010672.s007.docx]

S6 Table: Sequencing runs performed during this study.

*Samples tested to assess the entire ChIP-seq workflow.

These reads were neither included in the downstream data analysis nor submitted to NCBI.

| Sequencing | Sample | Replicate | Run # | Data yield |
| --- | --- | --- | --- | --- |
| RNA-Seq | ND3-pQE80L | R1 | 1 | 702.8 Mbp |
|  |  | R2 | 2 | 792.12 Mbp |
|  |  | R3 | 3 | 917.01 Mbp |
|  | ND3-pQE80L-*appY* | R1 | 1 | 723.02 Mbp |
|  |  | R2 | 2 | 774.54 Mbp |
|  |  | R3 | 3 | 942.25 Mbp |
| ChIP-Seq | ND3-pQE80L | R0* | 4 | 306.40 Mbp |
|  | ND3-pQE80L-*appY* | R0* | 4 |  |
|  |  | R1 | 5 |  |
|  |  | R2 | 6 |  |
|  |  | R3 | 6 |  |
|  | ND3-pQE80L-*appY-*3Flag | R0* | 4 |  |
|  |  | R1 | 5 |  |
|  |  | R2 | 6 |  |
|  |  | R3 | 6 |  |
|  | ND3-pQE80L-*appY_K170E_-*3Flag | R1 | 5 |  |
|  |  | R2 | 6 |  |
|  |  | R3 | 6 |  |
