# Supplementary material for: The prophage-encoded transcriptional regulator AppY has pleiotropic effects on E. coli physiology
Source: PLoS Genet. 2023 Mar 17;19(3):e1010672. doi: 10.1371/journal.pgen.1010672 (PMC10057817; doi:10.1371/journal.pgen.1010672)
Supplement: S7 Table — (DOCX) [file pgen.1010672.s008.docx]

S7 Table: Rockhopper mapping statistics (12,13)

| Construct | pQE80L | | | pQE80L-*appY* | | |
| --- | --- | --- | --- | --- | --- | --- |
| Replicate | R1 | R2 | R3 | R1 | R2 | R3 |
| Total reads | 4541549 | 5124029 | 5914850 | 4637874 | 5010046 | 6094302 |
| Successfully aligned reads | 86% | 92% | 91% | 98% | 98% | 98% |
| Aligning (sense) to protein-coding genes | 54% | 83% | 76% | 88% | 86% | 86% |
| Aligning (antisense) to protein-coding genes | 1% | 1% | 1% | 1% | 1% | 1% |
| Aligning (sense) to rRNA | 36% | 3% | 13% | 2% | 2% | 2% |
| Aligning (antisense) to rRNA | 4% | 0% | 0% | 0% | 0% | 0% |
| Aligning (sense) to tRNA | 0% | 1% | 0% | 0% | 0% | 0% |
| Aligning (antisense) to tRNA | 0% | 0% | 0% | 0% | 0% | 0% |
| Aligning (sense) to miscellaneous RNA | 2% | 5% | 3% | 3% | 4% | 4% |
| Aligning (antisense) to miscellaneous RNA | 0% | 0% | 0% | 0% | 0% | 0% |
| Aligning to unannotated region | 4% | 7% | 6% | 5% | 6% | 6% |
