# Supplementary figures and images for: The prophage-encoded transcriptional regulator AppY has pleiotropic effects on E. coli physiology
Source: PLoS Genet. 2023 Mar 17;19(3):e1010672. doi: 10.1371/journal.pgen.1010672 (PMC10057817; doi:10.1371/journal.pgen.1010672)

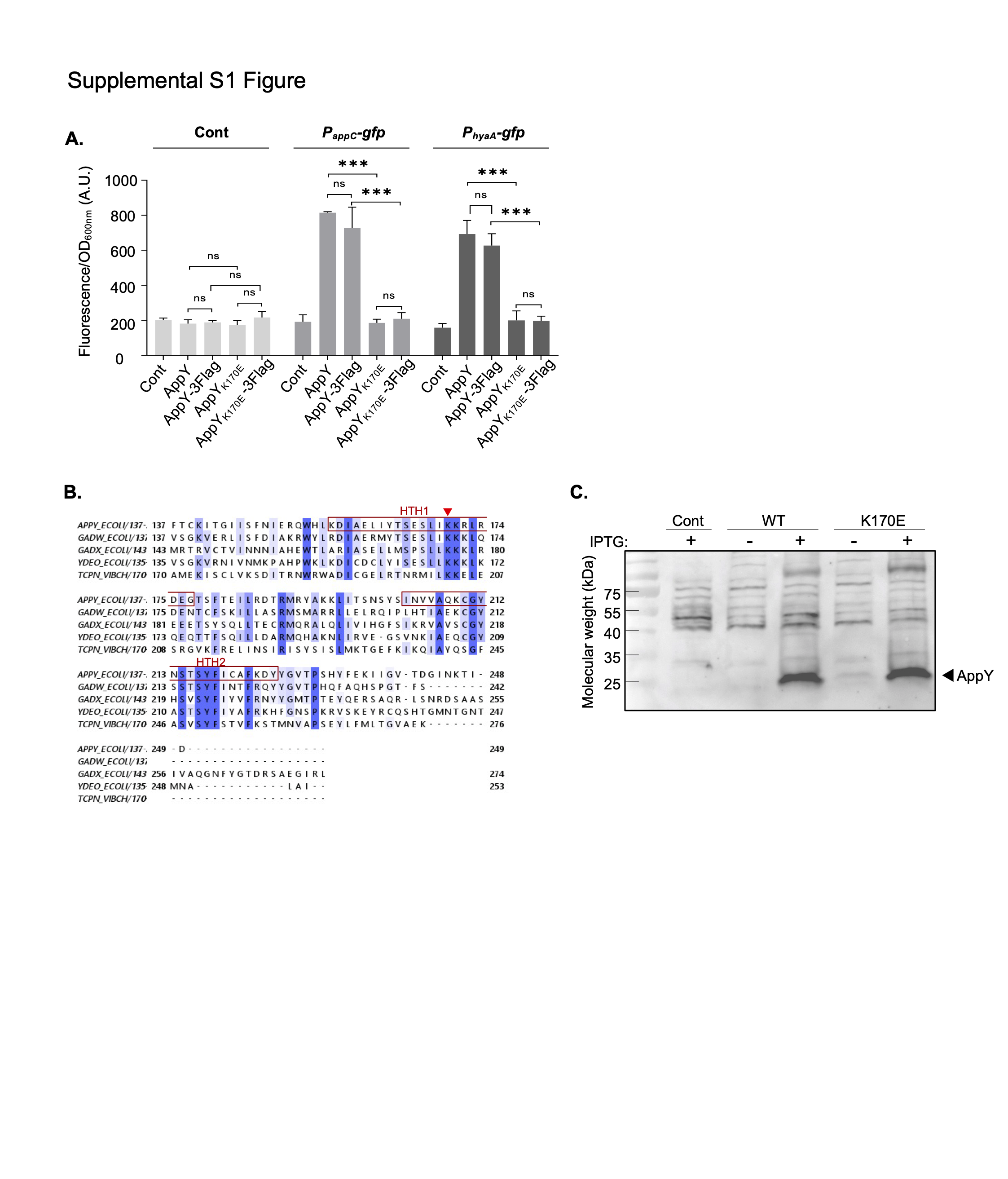

Supplement: S1 Fig — A. Functionality of the different AppY constructs used in this study. pQE80L and pQE-appYWT or pQE-appYK170E, with or without the 3-Flag tag were co-transformed in MG1655 ΔrpoS with the pUA66 empty vector (Cont) or the transcriptional fusions PappC-gfp and PhyaA-gfp. Cells were grown in LB at 37°C with 0.05 mM IPTG during 10 hours. Activity of the fusions was determined as described in Materials and Methods. The mean of 3 replicates is presented here and the standard deviation (SD) is indicated by the error bars. A.U., arbitrary units. Statistical significance calculations were performed using two-way ANOVA with Tukey’s multiple comparisons test (ns, not significant; ***, p-value < 0.001). B. Alignment of AppY C-terminal domain with other proteins from the AraC family. Sequence alignment was made using Jalview (12). AppY Helix-Turn-Helix motifs 1 and 2 are boxed in red. The intensity of the blue color reflects the residue conservation. The K170 residue mutated in this study is indicated by a red arrow. C. AppYWT and AppYK170E production. pQE80L empty vector (Cont) or containing appY (WT) or appY mutant (K170E) were transformed in MG1655 ΔrpoS. Cells were grown at 37°C in LB to OD600 ∼ 0.6 and 0.05 mM IPTG was added for 1 hour. AppY levels were analyzed by Western blotting using an anti-AppY antiserum. (TIF) [file pgen.1010672.s009.tif]

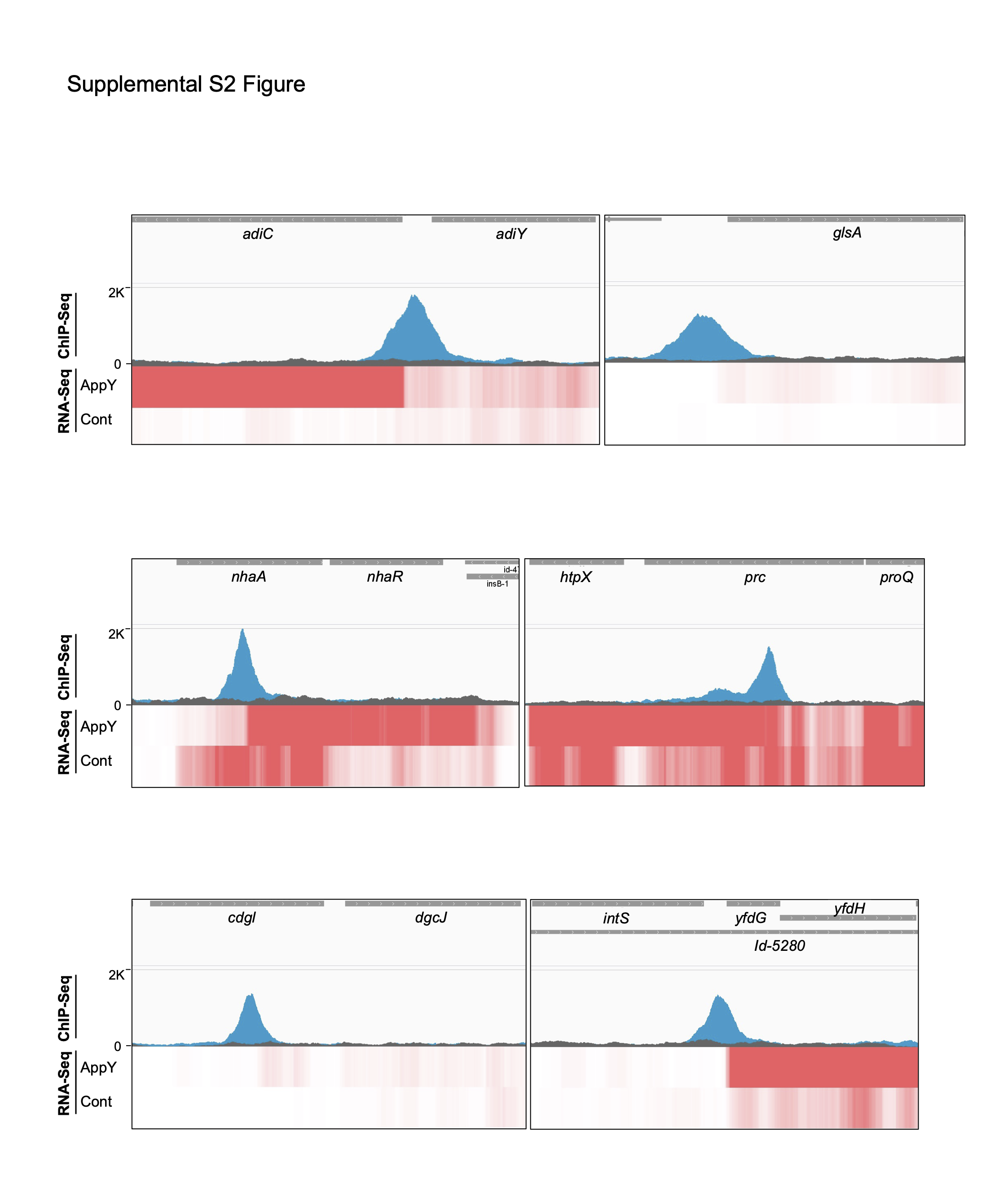

Supplement: S2 Fig — The genetic loci, the ChIP-Seq data obtained with AppY-3Flag and AppYK170E-3Flag and the gene expression profiles obtained by RNA-Seq with AppY or the vector control are shown from the top to the bottom. Peaks observed in ChIP-Seq with AppYWT (blue) or AppYK170E (black) are superimposed for comparison; the intensity of the red color in RNA-Seq panels represents the number of raw counts per gene. The data are representative of three independent experiments. (TIF) [file pgen.1010672.s010.tif]

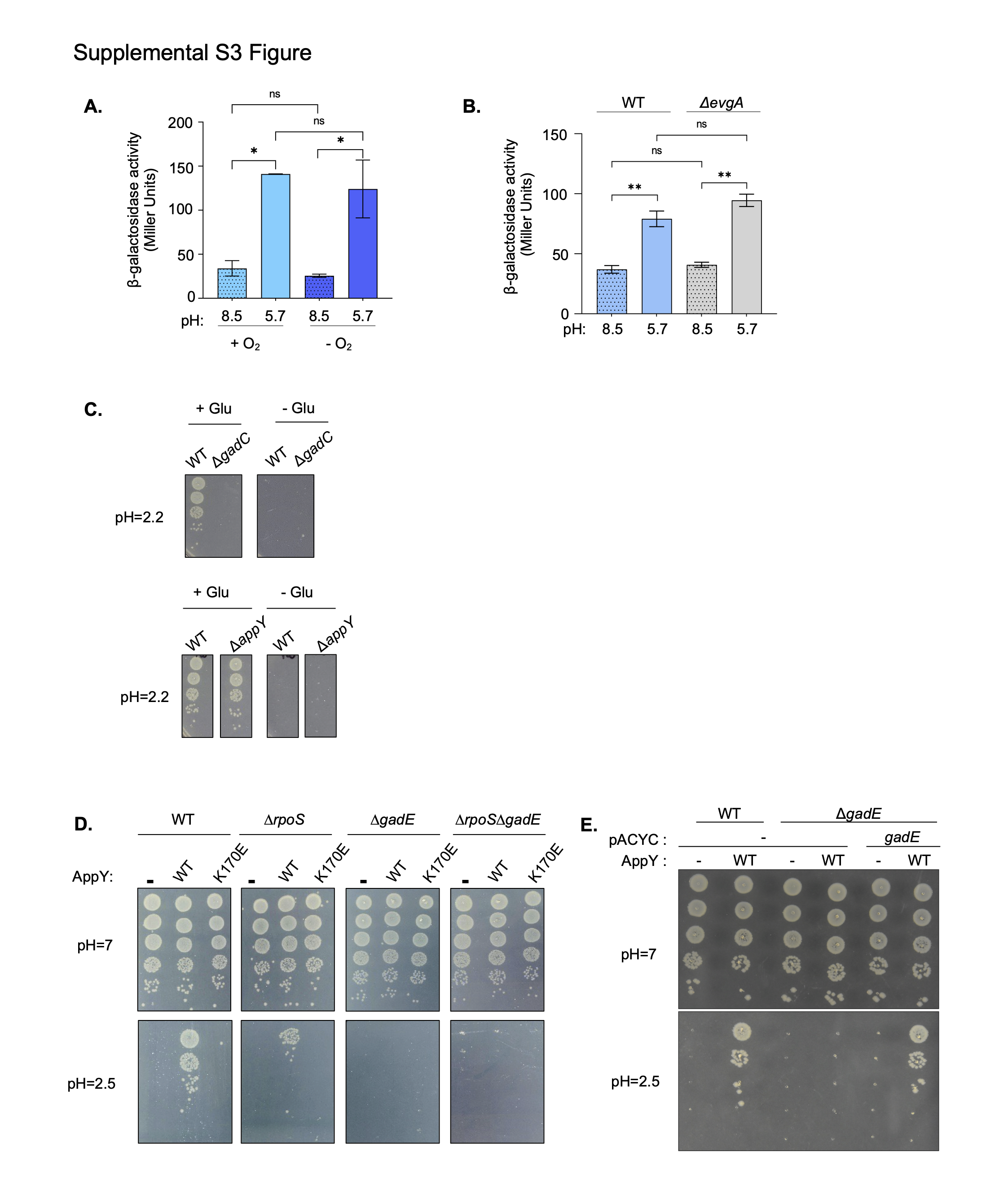

Supplement: S3 Fig — A. Strains carrying a chromosomal appY-lacZ translational fusion were cultured ON in LBK pH = 7, diluted 1:1000 into LB pH = 8.5 (dotted line) or 5.7 (plain) and incubated at 37°C in aerobic (light blue) or anaerobic (dark blue) conditions. Cultures were grown until an OD600 ∼ 0.4. The activity of the appY fusion was determined as described using the Miller assay (13). Data are means +/- standard deviation (n = 3). Statistical significance calculations were performed using one-way ANOVA with Tukey’s multiple comparisons test (ns, not significant; *, p-value < 0.05; **, p-value < 0.01). B. Experiments were performed as described in A with a WT (blue) and ΔevgA (grey) strains in aerobic conditions C. Strains BW25113 (WT) and ΔgadC or MG1655 (WT) and ΔappY, were grown in LB plus 0.4% glucose at 37°C for 22 hours. Cultures were diluted 1:1000 into EG medium pH = 2.2 and grown with or without sodium glutamate for 4 hours. Cells were serially diluted and 10 𝜇l of cultures were spotted on LB plate incubated at 37°C. D. AppY overproduction confers resistance to acid stress. MG1655 WT, ΔrpoS, ΔgadE or ΔgadE ΔrpoS strains transformed with pQE80L (-), pQE-appYWT (WT) or pQE-appYK170E (K170E) were grown to OD600 = 1 in LB broth (pH 7.0) with 1 mM IPTG. Cells were diluted 40-fold into LB broth (pH 2.5) and incubated for 1 h at 37°C. Cells were serially diluted and 10 μl of cultures were spotted on LB plate incubated at 37°C. E. gadE expressed under its own promotor restores cell survival when AppY is overproduced. Strains MG1655 and ΔgadE were co-transformed with pQE80L (-) or pQE-appYWT (WT) and pACYC184 (-) or pACYC184-gadE. The experiment was carried out as described in S3.D. (TIF) [file pgen.1010672.s011.tif]

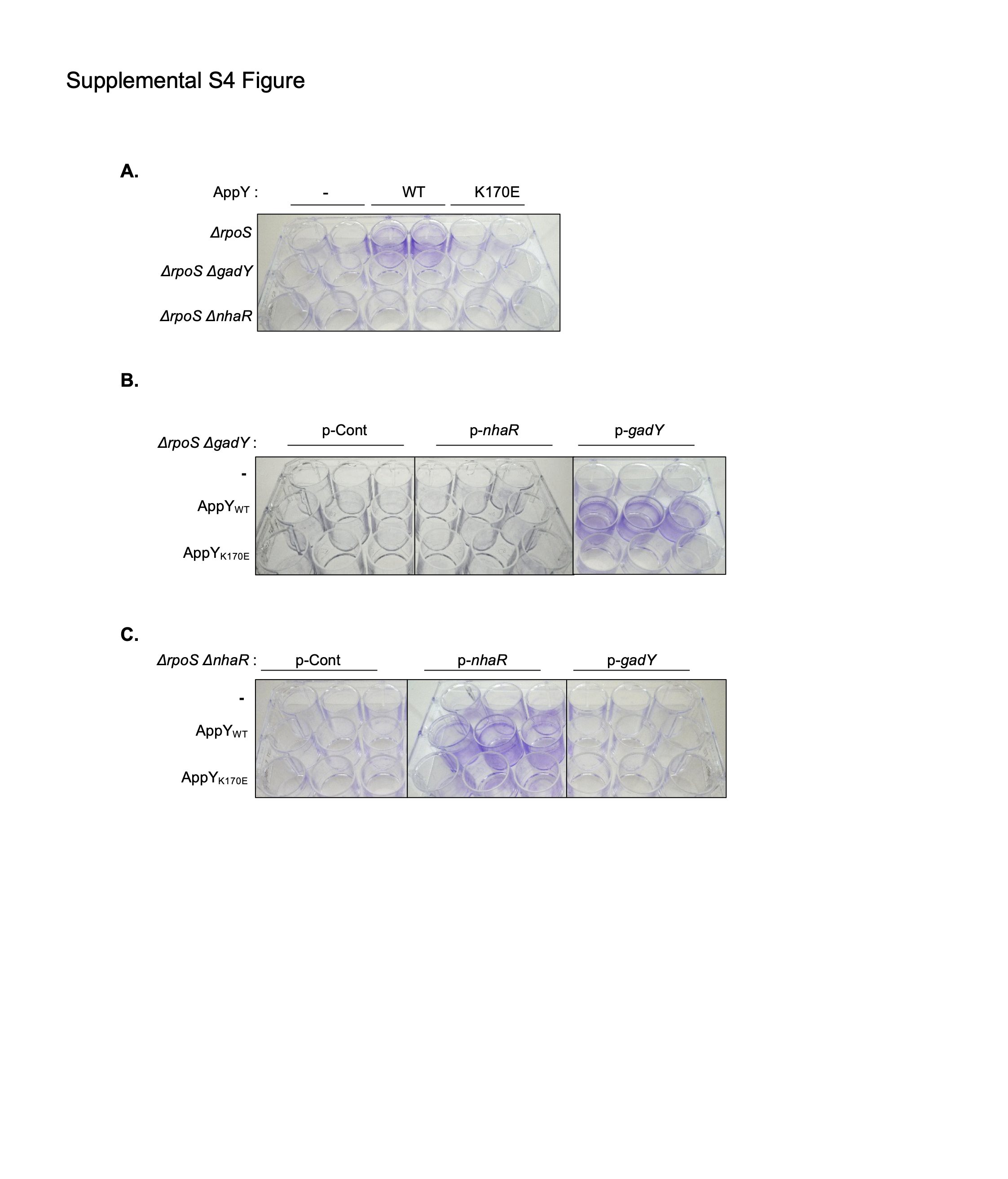

Supplement: S4 Fig — The indicated strains were grown in LB plus 0.5 mM IPTG at 30°C without shaking for 24 hours. Biofilm was visualized using crystal violet staining. A. Biofilm formation dependent on NhaR and GadY. MG1655 ΔrpoS, MG1655 ΔrpoS ΔgadY or MG1655 ΔrpoS ΔnhaR were transformed with the pQE80L empty vector (-) or containing appY (WT) or appY mutant (K170E). B. Complementation of MG1655 ΔrpoS ΔgadY with gadY and nhaR expressed under their own promoter. MG1655 ΔrpoS ΔgadY strain was co-transformed with a pQE80L construct (empty vector (-), containing appY (WT) or appY mutant (K170E)) and a pACYC184 construct (empty vector (p-Cont), containing nhaR (p-nhaR) or gadY (p-gadY)). C. Complementation of MG1655 ΔrpoS ΔnhaR with gadY and nhaR expressed under their own promoter. MG1655 ΔrpoS ΔnhaR strain was co-transformed with a pQE80L construct (empty vector (-), containing appY (WT) or appY mutant (K170E)) and a pACYC184 construct (empty vector (p-Cont), containing nhaR (p-nhaR) or gadY (p-gadY)). (TIF) [file pgen.1010672.s012.tif]

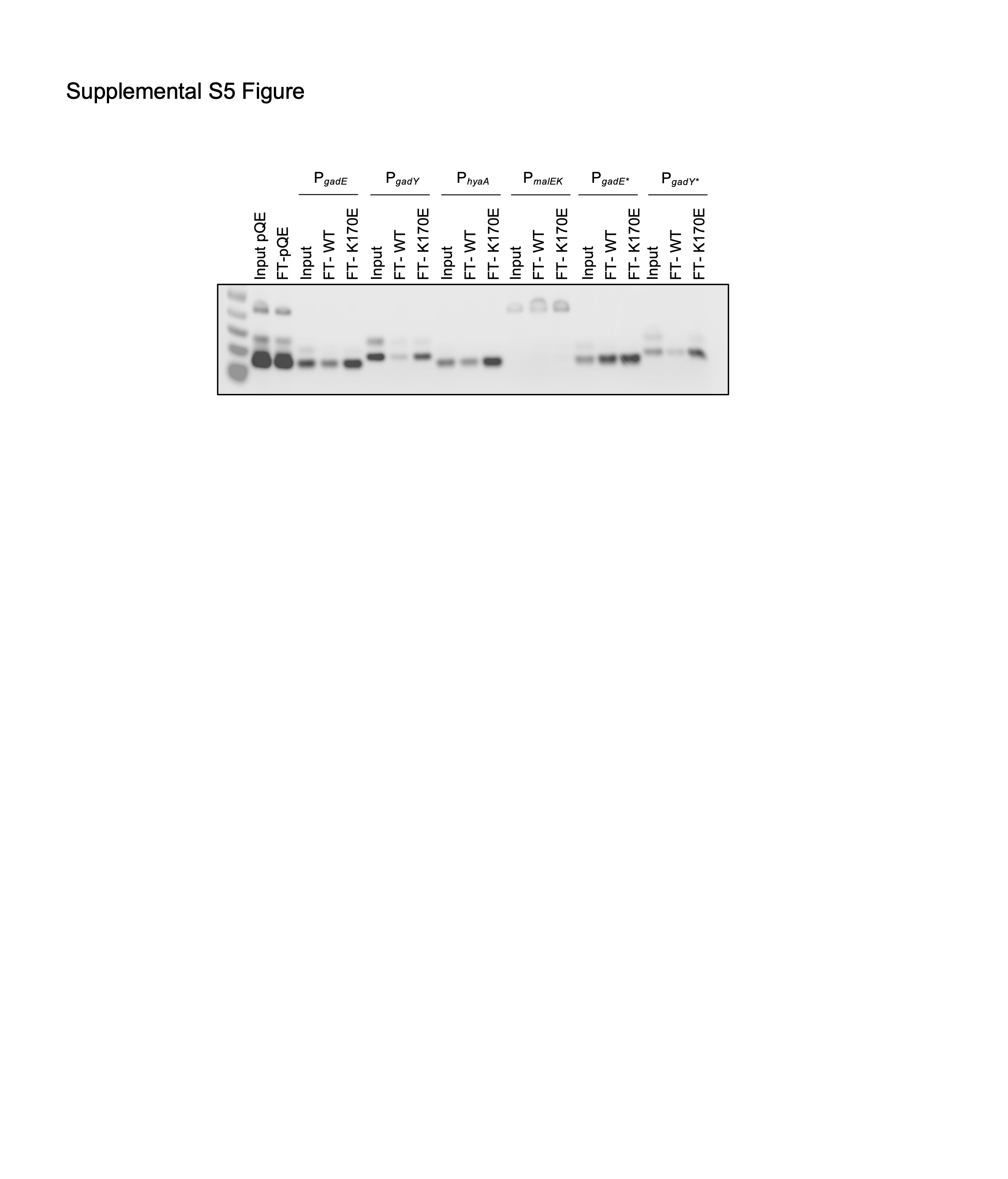

Supplement: S5 Fig — A solution of lysis buffer containing 500 ng of DNA was equally distributed in tubes containing the beads previously incubated with AppY-3Flag or AppYK170E-3Flag. Unspecific DNA binding to the beads, was checked by pooling all the DNA fragments and incubate them with the beads treated with a lysate containing only the pQE empty vector. To estimate the initial amount of DNA in our samples, 10 μl was loaded on a 1.5% TBE gel (Input). After DNA incubation with the beads, 10 μl of flowthrough (FT) was loaded on the same gel to estimate the amount of unbound DNA. Gel was scanned using a FLA500 (Fuji) scanner (excitation wavelength: 635 nm (800 V scanning intensity); emission wavelength: 665). (TIF) [file pgen.1010672.s013.tif]
